# Laparoscopic vs Open Distal Gastrectomy With D2 Lymphadenectomy for Clinical T4a Gastric Cancer: The UMC-UPPERGI-01 Randomized Clinical Trial

**DOI:** 10.1001/jamasurg.2025.4929

**Published:** 2025-11-12

**Authors:** Tran Quang Dat, Dang Quang Thong, Doan Thuy Nguyen, Nguyen Viet Hai, Tran Duy Phuoc, Nguyen Vu Tuan Anh, Nguyen Hoang Bac, Vo Duy Long

**Affiliations:** 1 Department of Gastrointestinal Surgery, University Medical Center, University of Medicine and Pharmacy at Ho Chi Minh City, Ho Chi Minh City, Vietnam; 2 Department of General Surgery, Faculty of Medicine, University of Medicine and Pharmacy at Ho Chi Minh City, Ho Chi Minh City, Vietnam

## Abstract

**Importance:**

No prospective trial on laparoscopy has been specifically designed for clinical T4a gastric cancer (GC); evidence of the safety and efficacy of laparoscopy for T4a GC is currently insufficient.

**Objective:**

To compare the short-term surgical outcomes between laparoscopic distal gastrectomy (LDG) and open distal gastrectomy (ODG) with D2 lymph node dissection for patients with clinical T4a GC.

**Design, Setting, and Participants:**

The UMC-UPPERGI-01 randomized clinical trial (RCT) was a single-center, open-label, noninferiority RCT conducted at a tertiary hospital in Ho Chi Minh City, Vietnam, among patients with clinical T4a GC. The study was conducted from June 2020 to April 2025. Interim and short-term results analyses were conducted in October 2023 and June 2025, respectively.

**Interventions:**

Patients were randomly assigned 1:1 to undergo LDG or ODG with D2 lymph node dissection as the full analysis set of the UMC-UPPERGI-01 trial. The procedures were performed exclusively by qualified surgeons.

**Main Outcomes and Measures:**

Short-term outcomes included surgical results, pathological characteristics, 30-day postoperative morbidity and mortality (Clavien-Dindo classification), and postoperative recovery parameters.

**Results:**

A total of 208 patients who underwent curative-intent distal gastrectomy with D2 lymphadenectomy were included in the full analysis set, with 104 patients in each group. There was no crossover between the 2 groups. Patients in the LDG group had a mean (SD) age of 61.1 (10.0) years and included 25 female patients (24.0%). Patients in the ODG group had a mean (SD) age of 60.0 (10.7) years and included 29 female patients (27.9%). No significant differences were found between the LDG and ODG groups in 30-day morbidity and mortality, including rates of any postoperative complication (22.1% vs 21.2%; *P* = .87) and severe complications (2.9% vs 3.8%; *P* > .99). The LDG group showed nonsignificant differences regarding surgical complications (21.2% vs 16.4%; *P* = .37) and general complications (3.9% vs 10.6%; *P* = .11) compared to the ODG group. The LDG group had significantly longer mean (SD) operative time (220.0 [42.4] minutes vs 153.7 [36.7] minutes; *P* < .001) and higher median (IQR) blood loss (80 [50-145] mL vs 50 [30-100] mL; *P* = .003). There were no significant differences in terms of time to the first flatus, time to oral tolerance, length of postoperative hospital stay, and time from surgery to initiation of adjuvant chemotherapy. Comorbidity was identified as an independent risk factor for postoperative complications in multivariate analysis (odds ratio, 2.42; 95% CI, 1.11-5.30; *P* = .03).

**Conclusions and Relevance:**

In the UMC-UPPERGI-01 noninferiority RCT, LDG showed comparable short-term outcomes to ODG for T4a GC. The results suggest that LDG can be recommended as an alternative to ODG by qualified surgeons for clinical T4a GC; ongoing follow-up will determine long-term oncologic outcomes.

**Trial Registration:**

ClinicalTrials.gov Identifier: NCT04384757

## Key Points

**Question:**

Is laparoscopic distal gastrectomy (LDG) noninferior to open distal gastrectomy (ODG) for clinical T4a gastric cancer (GC) in terms of short-term outcomes?

**Findings:**

In this randomized clinical trial (RCT) that included 208 patients with clinical T4a GC who underwent LDG or ODG, no significant differences were found in 30-day morbidity and mortality, including rates of any postoperative complication (22.1% vs 21.2%) and severe complications (2.9% vs 3.8%).

**Meaning:**

The results of this RCT provide robust evidence that LDG, when performed by qualified surgeons, may be a safe and appropriate treatment option for clinical T4a GC.

## Teaser

This noninferiority randomized clinical trial compares the short-term surgical outcomes between laparoscopic distal gastrectomy and open distal gastrectomy with D2 lymph node dissection for patients with clinical T4a gastric cancer.

## Introduction

Gastric cancer (GC) remains a major global health burden, ranking as one of the leading cancer-related mortalities worldwide.^1^ Recent advances in multimodal therapy have improved outcomes, but surgical resection continues to be a cornerstone for treating locally advanced GC (AGC).^2,3^ Standard surgical management includes gastrectomy with D2 lymphadenectomy, which has been demonstrated to provide oncological benefit in both Asian and Western populations.^4^

Laparoscopic gastrectomy has become widely accepted for early-stage GC in general practice.^5,6,7,8,9^ In the context of AGC, while landmark trials, such as CLASS-01,^10^ KLASS-02,^11^ and JLSSG0901,^12^ have established the noninferior long-term survival of laparoscopic distal gastrectomy (LDG) compared to open distal gastrectomy (ODG), these studies predominantly included patients with T2 or T3 tumors, with a small proportion of T4a cases. Of note, subgroup analysis of the JLSSG0901 trial^12^ in patients with T4a tumor suggested worse 5-year recurrence-free survival in those who underwent LDG compared to ODG. These tumors, defined by serosal invasion, are typically associated with more aggressive characteristics, such as large tumor size, high rates of lymph node (LN) metastasis, and peritumoral inflammatory reactions.^13,14,15^ These features lead to technical challenges in the dissection of LNs in the infrapyloric and suprapancreatic regions where there is increased risk of bleeding, pancreatic injury, and incomplete lymphadenectomy.^16,17^ Thus, the laparoscopic approach requires higher technical complexity than open surgery. Laparoscopic manipulation of the tumor and metastatic LNs in T4a tumors also raises concerns about the potential for tumor spillage, peritoneal seeding, and trocar site metastasis.^18,19,20^ These factors underscore the risks of elevated morbidity and possible compromise in oncological efficacy when performing laparoscopic resection for T4a GC.

Although retrospective studies have reported encouraging surgical outcomes and promising survival of LDG compared with ODG for T4a GC,^21,22,23,24,25^ these studies are limited by selection bias, lack of standardization in surgical techniques, and inadequate follow-up. Furthermore, regarding the benefits of laparoscopic surgery in postoperative complications, several studies demonstrated a lower complication rate in the LDG group compared to ODG,^26,27,28^ whereas other studies found no statistically significant differences in morbidity and mortality rates.^21,29,30^ Moreover, CLASS-01,^10^ KLASS-02,^11^ and JLSSG0901^12^ have not confirmed the effectiveness of LDG for the solely T4a subgroup. Therefore, the role of LDG in T4a GC remains controversial.

To date, no randomized clinical trial (RCT) has been specifically designed to evaluate the safety and efficacy of LDG for clinical T4a (cT4a) GC. We conducted this RCT to compare LDG and ODG with D2 lymphadenectomy in terms of early outcomes and long-term survival for patients with cT4a GC. In this article, we report the short-term surgical outcomes, including operative parameters, postoperative recovery, and early complications.

## Methods

### Study Design

This was a single-center, open-label, noninferiority RCT comparing LDG and ODG with D2 lymphadenectomy for patients with clinically diagnosed T4a GC. The study protocol was approved by the institutional review board of the University Medical Center at Ho Chi Minh City, Vietnam (26/HDDD-DHYD), on June 11, 2020, and was registered on ClinicalTrials.gov (NCT04384757). The protocol of this RCT had been published previously and is available in Supplement 1.^31^ Written informed consent was obtained from all patients before randomization. The trial was conducted in accordance with the Consolidated Standards of Reporting Trials (CONSORT) reporting guidelines. Data were collected and monitored by an independent data and safety monitoring committee (DSMC) organized by the Department of Scientific Research and Training of the University Medical Center at Ho Chi Minh City, Vietnam. The study was conducted from June 2020 to April 2025. Interim and short-term results analyses were conducted in October 2023 and June 2025, respectively.

### Patients

Eligible patients had cT4a GC suitable for curative resection by distal gastrectomy. The inclusion criteria were aged 18 to 80 years, with lower- or middle-third gastric adenocarcinoma, staged as clinical T4aN0-3M0 based on preoperative imaging, with Eastern Cooperative Oncology Group status of 0 to 1, and American Society Anesthesiology score of class I to III. Key exclusion criteria included neoadjuvant therapy; bulky LNs; previous gastric surgery; severe tumor-related complications, such as bleeding; perforation; other malignancies within 5 years; and pregnancy or severe conditions contraindicating laparoscopy (eTable 1 in Supplement 2).

### Sample Size

Sample size was estimated using the log-rank test for survival analysis in noninferiority trials with 3-year disease-free survival as the primary end point. A hazard ratio of 1.45 was used for the noninferiority margin (Δ_0_), type I error was set at .05 (1-sided) with 80% power, with a 1:1 allocation ratio. The total sample size required was 240 patients (120 patients in each group) after considering the 14% dropout rate.

### Preoperative Staging

Preoperative staging was performed exclusively by standardized thoracoabdominal contrast-enhanced computed tomography (CT) protocol. Specifically, all patients received 500 mL of water as an oral neutral contrast agent approximately 15 minutes prior to imaging. Clinical T4a stage was determined based on established radiologic criteria: nodular or irregular outer gastric wall, perigastric fat stranding, or hyperattenuating serosa sign.^32^ All CT scans were independently interpreted by at least 2 radiologists blinded to the treatment plan and subsequently reviewed in a multidisciplinary tumor board setting to determine final clinical staging and treatment plan.

### Surgical Standardization

All procedures were performed by 5 qualified surgeons, each with experience of more than 100 cases for both laparoscopic and open gastrectomy with D2 lymphadenectomy. Surgical techniques were standardized and validated by a procedural checklist. For quality assurance, the procedures were subject to periodic review by an independent DSMC, which included random video documentation of key operative steps to ensure technical consistency and protocol compliance.

### Randomization and Treatment Allocation

Patients were randomly assigned 1:1 to the LDG or ODG group using block randomization with random block sizes of 2, 4, or 6, generated by Stata version 16 (StataCorp). Allocation was concealed using sequentially numbered, opaque, sealed envelopes that were opened preoperatively. Due to the nature of surgical interventions, blinding of surgeons and patients was not possible. However, outcome assessors and data analysts remained blinded to group assignments.

### Surgical Procedures

In both groups, distal gastrectomy with D2 lymphadenectomy was performed according to the Japanese Gastric Cancer Treatment Guidelines. Peritoneal lavage cytology was conducted at the beginning of the surgery and repeated after gastric resection.

In the LDG group, totally laparoscopic gastrectomy and D2 lymphadenectomy were completed using a predefined, stepwise approach to ensure consistency and surgical quality. A key technical aspect of this trial was the adoption of the outermost layer-oriented medial approach for infrapyloric and suprapancreatic lymphadenectomy (stations 5, 6, 7, 8a, 9, and 11p).^16^ This approach involves dissecting the avascular space between the autonomic nerve sheaths and the lymphatic tissues, facilitating a safer and more efficient lymphadenectomy along major vessels.^16^ To minimize pancreatic injury during suprapancreatic dissection, a compressionless technique was used.^33^ Instead of directly compressing the pancreas, the assistant retracted the surrounding fatty tissues at the inferior border of the pancreas posteriorly to expose the operative field. Reconstruction was performed using either Billroth II or Roux-en-Y techniques. A prophylactic drainage was routinely placed.

In the open group, the surgical procedures were similar to those in the laparoscopic group but were conducted via laparotomy.

### Outcomes and Definitions

The short-term outcomes included operative characteristics, recovery parameters, and postoperative morbidity and mortality. Operative characteristics included operative time, estimated intraoperative blood loss, intraoperative vascular injury, major bleeding of more than 200 mL, conversion to ODG, and number of retrieved LNs. Recovery outcomes were time to first flatus, time to oral diet tolerance, postoperative length of hospital stay, and length from surgery to initiation of adjuvant chemotherapy. Morbidity and mortality events were recorded for those occurring within 30 days postoperatively and were classified using the Clavien-Dindo grading system.^34^ A severe complication was defined as grade IIIa or higher. Complications were diagnosed and recorded based on the list described by Baiocchi and colleagues.^35^ The definition and grading of postoperative pancreatic fistula (POPF) were based on the 2016 update of the International Study Group of Pancreatic Fistula.^36^

### Statistical Analysis

The population for short-term analysis was the actual treatment group, which included all patients who received curative gastrectomy, excluding patients who refused or withdrew or patients with intraoperatively diagnosed T4b or peritoneal metastasis (sT4b, sPM1). Patients who chose to switch surgical approaches (laparoscopic or open) after randomization were analyzed based on the actual treatment received rather than the assigned treatment.

All analyses were conducted using Stata version 17. Continuous variables were compared using either the *t* test or Mann-Whitney *U* test as applicable. Categorical variables were analyzed using the χ^2^ test or Fisher exact test. To evaluate factors influencing morbidity, binary logistic regression was used for both univariate and multivariate analysis. Variables were selected based on clinical relevance. *P* value tests were 2-tailed, and *P* < .05 was considered statistically significant.

## Results

### Study Flow and Analysis Population

Between June 2020 and April 2025, a total of 240 eligible patients with cT4a GC were randomized in a 1:1 ratio to undergo either LDG or ODG. Following randomization, 16 patients were excluded from each group. No patient in either group swapped from laparoscopic to open surgery or vice versa before resection (Figure). Ultimately, 208 patients (104 in each group) underwent curative-intent distal gastrectomy with D2 lymphadenectomy and were included in the full analysis set. All subsequent short-term outcome analyses were based on this population. Patients in the LDG group had a mean (SD) age of 61.1 (10.0) years and included 79 males (76.0%) and 25 females (24.0%). Meanwhile, patients in the ODG group had a mean (SD) age of 60.0 (10.7) years and included 75 males (72.1%) and 29 females (27.9%). Patient demographics and baseline characteristics were well balanced between the 2 groups (Table 1).

**Figure.**
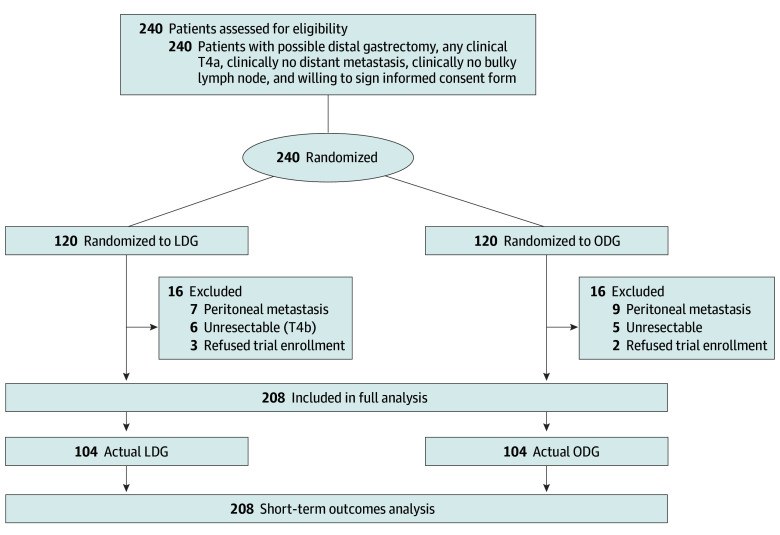
Consolidated Standards for Reporting Trials (CONSORT) Diagram LDG indicates laparoscopic distal gastrectomy; ODG, open distal gastrectomy.

**Table 1.** Patient Characteristics

Characteristic	No. (%)		*P* value
	LDG (n = 104)	ODG (n = 104)	
Age, mean (SD), y	61.1 (10.0)	60.0 (10.7)	.44
Sex			
Female	25 (24.0)	29 (27.9)	.53
Male	79 (76.0)	75 (72.1)	
BMI, mean (SD)^a^	21.6 (2.6)	21.4 (3.2)	.37
ASA-PS			
I	3 (2.9)	6 (5.8)	.58
II	45 (43.3)	41 (39.4)	
III	56 (53.8)	57 (54.8)	
ECOG status			
0	67 (64.4)	60 (57.7)	.32
1	37 (35.6)	44 (42.3)	
Hypertension	36 (34.6)	32 (30.8)	.55
Diabetes	13 (12.5)	6 (5.8)	.09
Cardiovascular disease	20 (19.2)	11 (10.6)	.08
Chronic lung disease	17 (16.4)	12 (11.5)	.32
Chronic hepatic disease	9 (8.7)	7 (6.7)	.60
Chronic kidney disease	2 (1.9)	3 (2.9)	>.99
Previous stroke	0	2 (1.9)	.50
History of upper abdominal surgery	2 (1.9)	0	.50
Hemoglobin, mean (SD), g/dL	12.68 (2.34)	12.68 (2.51)	.75
Albumin, mean (SD), g/dL	40.5 (6.5)	39.9 (6.4)	.86
Protein, mean (SD), g/dL	69.6 (7.4)	69.1 (9.2)	.91
Anemia	30 (28.9)	32 (30.8)	.76
Gastric outlet obstruction	29 (27.9)	31 (29.8)	.76
Tumor location			
Lower third	91 (87.5)	91 (87.5)	.91
Middle third	9 (8.7)	10 (9.6)	
Lower and middle third	4 (3.8)	3 (2.9)	
cN stage			
cN0	29 (27.9)	32 (30.8)	.65
cN+	75 (72.1)	72 (69.2)	

Abbreviations: ASA-PS, American Society of Anesthesiologists Physical Status; BMI, body mass index; cN, clinical lymph node; ECOG, Eastern Cooperative Oncology Group; LDG, laparoscopic distal gastrectomy; ODG, open distal gastrectomy.

SI conversion factors: To convert hemoglobin, albumin, and protein from g/dL to g/L, multiply by 10.

^a^ Calculated as weight in kilograms divided by height in meters squared.

### Surgical Outcomes

All patients underwent distal gastrectomy with D2 lymphadenectomy according to the study protocol. The mean (SD) operative time was significantly longer in the LDG group than in the ODG group (220.0 [42.4] minutes vs 153.7 [36.7] minutes; *P* < .001). Median (IQR) intraoperative blood loss was also significantly higher in the LDG group (80 [50-145] mL vs 50 [30-100] mL; *P* = .003). In the LDG group, 1 patient (1.0%) was converted to open surgery due to extensive suprapancreatic nodal involvement, as the surgeon deemed laparoscopic dissection unsafe.

No significant differences were observed in the incidence of intraoperative complications between the 2 groups. The rates of major bleeding (≥200 mL) and vascular injury were comparable (major bleeding: 18.3% vs 14.4%; *P* = .45; vascular injury: 2.9% vs 3.9%; *P* > .99). No intraoperative mortality occurred in either group. The types of reconstruction differed significantly between the 2 groups, with a higher proportion of Roux-en-Y anastomosis performed in the LDG group (31.7% vs 11.5%; *P* = .001). The mean lengths of proximal and distal resection margins were similar between groups (Table 2).

**Table 2.** Surgical Outcomes

Outcome	No. (%)		*P* value
	LDG (n = 104)	ODG (n = 104)	
Operating time, mean (SD), min	220 (42.4)	153.7 (36.7)	<.001
Blood loss, median (IQR), mL	80 (50-145)	50 (30-100)	.003
Concurrent resection	8 (7.7)	6 (5.8)	.78
Intraoperative bleeding >200 mL	19 (18.3)	15 (14.4)	.45
Intraoperative blood transfusion	0	0	>.99
Intraoperative injury	3 (2.9)	4 (3.9)	
Splenic injury	0	2 (1.9)	>.99
Arterial injury	2 (1.9)	1 (1.0)	
Venous injury	1 (1.0)	1 (1.0)	
Gastrointestinal injury	0	0	
Conversion to open surgery	1 (1.0)	NA	NA
Proximal margin, mean (SD), cm	6.2 (1.7)	6.1 (1.9)	.31
Distal margin, mean (SD), cm	3.2 (1.8)	3.1 (1.7)	.54
Type of reconstruction			
Billroth II	71 (68.3)	92 (88.5)	.001
Roux-en-Y	33 (31.7)	12 (11.5)	

Abbreviations: LDG, laparoscopic distal gastrectomy; NA, not applicable; ODG, open distal gastrectomy.

### Pathological Characteristics

Mean tumor size, macroscopic type, pathologic T and N stages, pTNM staging, lymphovascular invasion and perineural invasion, and pathological differentiation status were not significantly different between the 2 groups. The mean (SD) tumor size was 5.1 (1.8) cm in the LDG group and 5.2 (2.1) cm in the ODG group (*P* = .86). The median (IQR) number of retrieved LNs was 32.5 (25-42) in the LDG group and 33.0 (26-42) in the ODG group (*P* = .53). The rate of LN metastasis was comparable (74.0% vs 76.9%). Positive resection margin was found in 1 patient (1%) in the LDG group and 2 patients (1.9%) in the ODG group, while peritoneal lavage cytology was positive in 6 patients (5.8%) in the LDG group and 5 patients (4.8%) in the ODG group (Table 3).

**Table 3.** Pathological Characteristics

Characteristic	No. (%)		*P* value
	LDG (n = 104)	ODG (n = 104)	
Tumor size, mean (SD), cm	5.1 (1.8)	5.2 (2.1)	.86
Macroscopic type^a^			
1	3 (2.9)	7 (6.7)	.55
2	48 (46.2)	49 (47.1)	
3	47 (45.2)	41 (39.4)	
4	6 (5.8)	7 (6.7)	
No. of retrieved lymph nodes, median (IQR)	32.5 (25-42)	33 (26-42)	.53
R1 category	7 (6.7)	7 (6.7)	
Positive cytology	6 (5.8)	5 (4.8)	>.99
Positive resection margin	1 (1.0)	2 (1.9)	
pT stage			
T1	1 (1)	3 (2.9)	.61
T2	7 (6.7)	7 (6.7)	
T3	26 (25)	20 (19.2)	
T4a	70 (67.3)	74 (71.2)	
pN stage			
N0	27 (26)	24 (23.1)	.36
N1	18 (17.3)	20 (19.2)	
N2	25 (24)	20 (19.2)	
N3a	23 (22.1)	19 (18.3)	
N3b	11 (10.6)	21 (20.2)	
TNM stage			
I	4 (3.8)	6 (5.8)	.73
IIA	11 (10.6)	8 (7.7)	
IIB	18 (17.3)	15 (14.4)	
IIIA	34 (32.7)	35 (33.7)	
IIIB	21 (20.2)	17 (16.4)	
IIIC	10 (9.6)	18 (17.3)	
IV (CY (+)	6 (5.8)	5 (4.8)	
Lymphovascular invasion	29 (27.9)	36 (34.6)	.30
Perineural invasion	61 (58.7)	60 (57.7)	.89
Differentiated status			
Differentiated	45 (43.3)	46 (44.2)	.89
Undifferentiated	59 (56.7)	58 (55.6)	

Abbreviations: CY, peritoneal lavage cytology; LDG, laparoscopic distal gastrectomy; ODG, open distal gastrectomy; TNM, tumor, node, metastasis.

^a^ Macroscopic type in accordance with Japanese classification of gastric carcinoma.

### Postoperative Morbidity and Mortality

The overall 30-day postoperative morbidity was similar between the LDG and ODG groups (22.1% vs 21.2%; *P* = .87). Most complications were minor, classified as Clavien-Dindo grade I or II. The incidence of major complications (Clavien-Dindo grade III or higher) was also equivalent (2.9% vs 3.8%; *P* > .99). Rates of specific complications were comparable, including POPF (7.7% vs 3.9%; *P* = .37), intraluminal bleeding (0% vs 1.9%; *P* = .50), and intra-abdominal abscess (2.9% vs 3.8%; *P* > .99). There was no case of anastomotic leakage, duodenal stump leak, or intra-abdominal hemorrhage in either group.

Regarding the pattern of complications, the LDG group showed nonsignificant differences regarding surgical complications, including wound infection, paralytic ileus, and POPF (21.2% vs 16.4%; *P* = .37), and regarding general complications, such as pulmonary, cardiovascular, and systemic infectious events (3.9% vs 10.6%; *P* = .11), compared to the ODG group.

Reoperation was required in 2 patients (1.9%) only in the LDG group. One patient developed a grade C POPF, requiring open surgery for peripancreatic drainage. Unfortunately, this patient subsequently experienced decompensated multi-organ failure and died. The other patient experienced a refractory intra-abdominal abscess, requiring an open surgical intervention for drainage.

Thirty-day mortality occurred in 1 patient (1.0%) in the LDG group and 2 patients (1.9%) in the ODG group (*P* > .99). The death in the LDG group was the previously mentioned patient with severe pancreatic fistula. In the ODG group, 1 patient died of aspiration pneumonia and another died of septic shock secondary to gastrointestinal infection; neither case showed evidence of anastomotic or pancreatic leakage. Median (IQR) comprehensive complication index scores for patients with any postoperative complications were not significantly different between the groups (LDG: 12.2 [8.7-12.9] vs ODG: 20.9 [8.7-26.2]; *P* = .09) (Table 4).

**Table 4.** Postoperative Outcomes

Outcome	No. (%)		*P* value
	LDG (n = 104)	ODG (n = 104)	
Postoperative hospital stay, mean (SD), d	7.4 (2.8)	7.5 (1.9)	.13
Time to flatus, mean (SD), d	3.0 (1.0)	3.1 (1.2)	.95
Time to oral diet tolerance, mean (SD), d	2.4 (1.1)	2.8 (1.3)	.05
Time to adjuvant chemotherapy, median (IQR), d	33 (31-37.5)	34 (31-41)	.30
Postoperative drainage amylase, median (IQR), U/L	203.5 (85.1-586.8)	197 (84-443)	.54
POD3 amylase drainage >3 × upper limit of normal serum amylase	45 (43.3)	40 (38.5)	.48
Postoperative complication			
Overall morbidity	23 (22.1)	22 (21.2)	.87
Anastomotic leakage	0	0	>.99
Anastomotic stricture	2 (1.9)	2 (1.9)	>.99
Duodenal stump leakage	0	0	>.99
Pancreatic fistula	8 (7.7)	4 (3.9)	.37
Paralytic ileus	10 (9.6)	5 (4.9)	.28
Pancreatitis	0	0	>.99
Chyle leak	1 (1.0)	1 (1.0)	>.99
Small-bowel obstruction	0	2 (1.9)	.50
Intra-abdominal bleeding	0	0	>.99
Intraluminal bleeding	0	2 (1.9)	.50
Intra-abdominal abscess	3 (2.9)	4 (3.8)	>.99
Wound infection	5 (4.8)	1 (1.0)	.21
Cardiovascular complications	0	3 (2.9)	.25
Pulmonary complications	2 (1.9)	6 (5.8)	.28
Systemic infection	2 (1.9)	4 (3.9)	.68
Reoperation	2 (1.9)	0	.50
General complications	4 (3.9)	11 (10.6)	.11
Surgical complications	22 (21.2)	17 (16.4)	.37
Clavien-Dindo classification, grade			
I	14 (13.5)	7 (6.7)	.33
II	6 (5.8)	11 (10.6)	
IIIa	1 (1.0)	2 (1.9)	
IIIb	1 (1.0)	0	
IVa	0	0	
V	1 (1.0)	2 (1.9)	
Major complication (Clavien-Dindo ≥IIIa)	3 (2.9)	4 (3.8)	>.99
CCI, median (IQR)			
For all analyzed population	0 (0-0)	0 (0-0)	.95
For patients with any complications	12.2 (8.7-12.9)	20.9 (8.7-26.2)	.09

Abbreviations: CCI, Comprehensive Complication Index; LDG, laparoscopic distal gastrectomy; ODG, open distal gastrectomy; POD3, postoperative day 3.

SI conversion factor: To convert amylase from U/L to µkat/L, multiply by 0.0167.

### Postoperative Recovery

Postoperative recovery parameters were equivalent between the 2 groups. There were no significant differences between the LDG and ODG groups in term of mean (SD) time to the first flatus (3.0 [1.0] days vs 3.1 [1.2] days; *P* = .95), time to oral diet tolerance (2.4 [1.1] days vs 2.8 [1.3] days; *P* = .05), and length of postoperative hospital stay (7.4 [2.8] days vs 7.5 [1.9] days; *P* = .13). Additionally, the median (IQR) time from surgery to initiation of adjuvant chemotherapy was also comparable (33 [31-37.5] days vs 34 [31-41] days; *P* = .30) (Table 4).

### Risk Factors Related to Postoperative Morbidity

In the multivariate analyses, only the presence of a comorbidity factor was identified as an independent risk factor for postoperative complications (odds ratio [OR], 2.42; 95% CI, 1.11-5.30; *P* = .03). In particular, the surgical approach (LDG vs ODG) was not an independent predictor of postoperative morbidity (OR, 0.85; 95% CI, 0.44-1.63; *P* = .62) (eTable 2 in Supplement 2).

## Discussion

While LDG is well established for early and locally advanced GC, high-quality evidence for its application to serosa-invasive (T4a) GC is still lacking. Prior landmark trials underrepresented this high-risk subgroup, and a subgroup analysis from the JLSSG0901 trial^37^ even raised concerns about the oncologic safety of LDG in this cohort. This is the first RCT designed specifically to address this critical evidence gap by exclusively enrolling patients with cT4a GC.

Despite a longer operative time and slightly higher blood loss compared to ODG, LDG with standardized surgical techniques yielded comparable morbidity, mortality, and postoperative recovery outcomes. Moreover, there were no significant differences in intraoperative complications and the number of retrieved LNs. Therefore, our study demonstrates that LDG with D2 lymphadenectomy is feasible and safe in experienced centers.

There were notable observations compared to previous RCTs in locally AGC, such as KLASS-02,^26^ CLASS-01,^38^ and JLSSG0901.^37^ Similar to these trials, the LDG group had significantly longer operative times compared to the ODG group. In contrast, blood loss was higher in the LDG group than in the ODG group, which differs from these trials. This issue could be explained by the complexity of lymphadenectomy, peritumoral inflammation and angiogenesis, and difficulty in bleeding control by laparoscopy. This discrepancy reflects the technical difficulty in laparoscopic dissection in T4a tumors, which may not be properly evaluated in prior trials due to the predominant proportion of T2 and T3 tumors.

Regarding early complications, while the KLASS-02 trial^26^ reported significantly lower morbidity in the laparoscopic group (16.6%) compared to open surgery (24.1%), our trial showed comparable morbidity rates between the 2 groups (22.1% vs 21.2%; *P* = .87), which was similar to the CLASS-01 and JLSSG0901 trials.^37,38^ Additionally, several retrospective studies in T4a GC demonstrated heterogeneous findings of postoperative complications.

For recovery outcomes, although previous studies demonstrated better postoperative recovery in laparoscopy compared to open,^26,38,39^ our study showed comparable results between the groups. Moreover, the time to initiation of adjuvant chemotherapy in our study was consistent with several previous retrospective studies,^25,40,41,42^ while other RCTs did not report this parameter.^12,26,38^ These findings suggested that postoperative recovery may be attributed to the standardized perioperative care, which adhered to Enhanced Reovery After Surgery (ERAS) protocol and predefined schedules for adjuvant therapy in both groups.

Taken together, these findings indicate that short-term outcomes of LDG vs ODG for T4a GC remained inconsistent. These discrepancies were likely due to the limited number of T4a GC cases in prospective trials and inherent limitations in retrospective studies, including selection bias, chronological bias, and a lack of standardization in surgical technique and perioperative care. Nevertheless, LDG was considered a safe and feasible option when performed by qualified surgeons. Importantly, by using rigorous inclusion criteria, standardized surgical protocols, and prospective data collection, our study provided additional evidence that supports the safety and feasibility of LDG for cT4a GC.

To overcome the technical difficulty and to achieve satisfactory surgical outcomes in LDG for T4a GC, technical standardization by qualified surgeons was strictly demanded. All procedures in this study adhered to the standard surgical checklist for ensuring adequate dissection and were performed by 5 senior surgeons who had experienced more than 100 standard open and laparoscopic gastrectomies with D2 lymphadenectomy. Thereby, our study achieved a 100% compliance rate of D2 LNs dissection in both groups.

POPF is a known risk of laparoscopic gastrectomy caused by inadvertent manipulation and thermal damage.^5,17,43,44,45^ Recognizing this challenge, our trial applied standardized techniques to minimize pancreatic injury and enhance precise dissection, including the outermost layer-oriented medial approach^16,46,47^ and pancreatic compressionless technique for dissection in the peripancreatic region.^33,48^ Consequently, we observed no significant difference of POPF rate, with only 1 grade C case in the LDG group. Moreover, this standardization likely contributed to the low rates of intraoperative complications and comparable morbidity between the LDG and ODG groups. Hence, these approaches should be recommended as a routine application when performing laparoscopic peripancreatic LNs dissection.

Our findings on the safety of LDG for cT4a GC should be interpreted in the context of current and evolving treatment paradigms including neoadjuvant therapy and robotic surgery. Although our trial reflects the up-front surgery approach, these results provide reassurance for applying LDG for T4a GC after neoadjuvant settings, where technical challenges may persist despite tumor downstaging. Regarding robotic surgery, while robotic platforms offer theoretical advantages for such complex cases, evidence of superiority over laparoscopy for AGC is inconclusive and mainly limited for early-stage GC.^49,50,51^ Furthermore, its adoption is limited by high cost and availability. By establishing a safety benchmark for laparoscopy in this high-risk population, our trial provides a crucial reference for both its application after neoadjuvant therapy and for future trials comparing it against emerging robotic techniques.

### Strengths and Limitations

Overall, the notable strength of this study is the robust evidence specifically for the application of LDG for cT4a GC, a subgroup underrepresented in previous trials. Results of this RCT could be beneficial for decision-making practice in the treatment of AGC. Nevertheless, this study has several limitations. First, as a single-center trial performed by expert surgeons at a high-volume institution, the findings’ generalizability to centers with less experience in advanced laparoscopic gastrectomy may be limited. Second, the reliance on preoperative CT for staging, which has limitations in diagnosing serosal invasion, led to a small proportion of pT1 or T2 tumors, potentially weakening the statistical power for T4a-specific comparisons.

## Conclusions

In conclusion, our findings in this noninferiority RCT demonstrate that LDG is both feasible and safe in the serosa-invasive subgroup when performed by qualified surgeons. This RCT fulfills a critical evidence gap regarding the application of LDG for locally advanced GC and supports the potential of this approach as an alternative for cT4a GC. The ongoing follow-up of this trial will determine whether LDG is noninferior to ODG regarding oncological survival, which is necessary for confirming its efficacy in the curative treatment of T4a GC.

## Notes

Supplement 1. Trial Protocol

Supplement 2. eTable 1. Inclusion and Exclusion Criteria eTable 2. Univariate and Multivariate Analysis for Predicting Postoperative Morbidity

Supplement 3. Data Sharing Statement
