# Supplementary material for: Laparoscopic vs Open Distal Gastrectomy With D2 Lymphadenectomy for Clinical T4a Gastric Cancer: The UMC-UPPERGI-01 Randomized Clinical Trial
Source: JAMA Surg. 2025 Nov 12;161(1):9–18. doi: 10.1001/jamasurg.2025.4929 (PMC12613089; doi:10.1001/jamasurg.2025.4929)
Supplement: Supplement 1. — Trial Protocol [file jamasurg-e254929-s001.pdf]

Protocol

Protocol for: Supplement to: Dat Tq, et al.

Laparoscopic versus Open Distal Gastrectomy with D2 Lymph Node Dissection For Clinical T4a Gastric Cancer: The UMC-UPPERGI-01 Randomized Clinical Trial.

This trial protocol has been provided by the authors to give readers additional information about the work.

Table of Contents

Table of Contents *2*

Summary *1*

1. Research background *6*

2. Objective *7*

3. Research design *7*

4. Study objects *9*

5. Diagnostic criteria for this study *11*

6. Outcome Measures *11*

7. Qualifications of the participated Surgeons *12*

8. End point and definition of related result determination........13

9. Standard trial proceedings........20

10. Data management *34*

11. Statistical Analysis *35*

12. Ethical approval *36*

REFERENCE *37*

Appendix 1: FLOW DIAGRAM FOR RCT LDG vs ODG *40*

Appendix 2: INFORMED CONSENT FORM *41*

Appendix 3: Ethical approval by Institutional Review Board, University Medical Center Ho Chi Minh city *47*

Laparoscopic versus Open Distal Gastrectomy with D2 Lymph Node Dissection For Clinical T4a Gastric Cancer The UMC-UPPERGI-01 Randomized Clinical Trial.

Bidding party: University Medical Center Ho Chi Minh city

Principle Investigator:

Prof. Vo Duy Long, M.D. Ph.D

Department of Gastro-intestinal Surgery, University Medical Center, University of Medicine and Pharmacy at Ho Chi Minh City, Ho Chi Minh City, Viet Nam

Address: No. 217 Hong Bang street, District 5, Ho Chi Minh City, Viet Nam.

Telephone: (+84-28) 3952 5656. Fax: (+84-28) 3950 6126

Summary

Scenario Title	**Laparoscopic versus Open Distal Gastrectomy with D2 Lymph Node Dissection For Clinical T4a Gastric Cancer: The UMC-UPPERGI-01 Randomized Clinical Trial**
Version	6
Sponsor	Vo Duy Long
Research Center	University Medical Center Ho Chi Minh city
Indications	Clinical T4a gastric adenocarcinoma with curative-intent distal gastrectomy (cT4a, N0/+, M0)
Purpose of research	To compare laparoscopic distal gastrectomy (LDG) with D2 lymphadenectomy to conventional open distal gastrectomy (ODG) in patients with clinically T4a gastric cancer (GC) regarding surgical safety

	and long-term oncologic outcomes
Research design	Single center, prospective, open-label, randomized controlled
Case grouping	Group A (Study Group): Laparoscopic distal gastrectomy (LDG) Group<br>Group B (Control Group): Open distal gastrectomy (ODG) Group
The basis for determining the sample size	This is a phase III, randomized controlled, non-inferiority trial, whose primary outcome measure is the 3-years disease-free survival (DFS). Patients with cT4aN0-3M0 suitable for distal gastrectomy with D2 lymph node (LNs) dissection will be randomly assigned in a 1:1 ratio to undergo either LDG or ODG. This analysis was based on an α of 0.05, a power of 80%, and hazard ratio (HR) of 1.45 as the non-inferiority margin (Δ_0), revealing that at least 103 patients would be necessary per group. Considering an expected dropout rate of 14%, it was determined that each group needed at least 120 patients, for a total of 240 cases.
Inclusion criteria	• Age 18–80 years.<br>• ECOG performance status of 0 or 1.<br>• ASA score of I–III.<br>• Clinical diagnosis with T4aN0-3M0 gastric adenocarcinoma suitable for curative resection by distal gastrectomy with D2 lymphadenectomy based on preoperative imaging.<br>• Willingness to participate the study and written informed consent.
Exclusion criteria	• Bulky LNs on preoperative findings.<br>• Previous gastric surgery<br>• Severe tumor-related complications such as bleeding or perforation.<br>• Prior chemotherapy or radiotherapy.<br>• Diagnosis of other malignancies within the past 5 years.<br>• Severe comorbidities or vulnerable conditions (eg,

	cognitive impairment, ongoing or planned pregnancy) contraindicating laparoscopy<br>• Participation in another clinical trial
Withdrawal criteria	● M1 tumor confirmed intraoperatively and did not received gastrectomy aimed to curative treatment.<br>● Patients intraoperatively confirmed as T4b and did not received gastrectomy<br>● Sudden severe complications during the preoperative period (intolerable surgery or anesthesia), which renders it unsuitable or unfeasible to implement the study treatment protocol as scheduled;<br>● Patients confirmed to require emergency surgery due to changes in the patient's condition after inclusion in this study;<br>● Patients who voluntarily quit or discontinue treatment for personal reasons at any stage after inclusion in this study;
Intervention	For patients who were assigned to LDG group:<br>● 5 trocars were used. The gastrocolic ligament was divided along the border of the transverse colon. ligating the left gastroepiploic vessels to remove group 4sb. The right gastroepiploic vein was divided and the right gastroepiploic and the inferior pyloric artery were vascularized and cut at their origin from the gastroduodenal artery, just above the pancreatic head, to dissect group 6.<br>● The dissection was continued along the hepatoduodenal ligament to removed group 5 and group 12a and along the common hepatic artery to remove group 8a and along the celiac axis to remove group 9.<br>● The left gastric vein was prepared and separately divided and then the left gastric artery was vascularized to remove group 7.<br>● The dissection was continued upward along the proximal branches of splenic vessels to remove group 11p and along the lesser

	curvature to remove group 1,3.<br>● The outermost layer-oriented medial approach and pancreatic compressionless techniques were standardized in all procedures.
Outcome Measures	Primary Outcome Measures:<br>● 3-year disease free survival (DFS).<br>Secondary Outcome Measures:<br>● Surgical outcomes: Operating time, Blood loss, intra-operative bleeding > 200ml, blood transfusion, intra-operative organ injury, conversion to open surgery, R1 resection rate, number of retrieved LNs<br>● Early complications: wound infections, intra-abdominal or intraluminal bleeding, fluid collection or abscess, anastomotic or duodenal stump leakage, anastomotic stenosis, pancreatic fistula, postoperative ileus, cardio-pulmonary complications<br>● Postoperative recovery: Postoperative hospital stay, Time to flatus, Time to oral tolerance, Time interval between surgery and adjuvant chemotherapy<br>● Late complications: intestinal obstruction related to adhesion, abdominal incision hernia, chronic wound infection<br>● Quality of life
Statistical considerati ons	● Outcomes will be analyzed using per-protocol (PP), and full analysis set (FAS) populations. Patients who crossover treatments preoperatively will be analyzed in the as-treated group (based on the actual procedure performed), while those converting from laparoscopic to open surgery intraoperatively remain in the laparoscopic group.<br>● Summary statistics are mean ± standard deviation or median (interquartile range: IQR) for continuous variables and frequency and percentage for categorical variables. Continuous variables are

	compared using t-tests or Mann–Whitney U tests. Categorical variables ared analyzed using chi-squared or Fisher's exact tests. Kaplan–Meier method is used to estimate OS and DFS, with comparisons between groups using the log-rank test. Hazard ratios with 95% is calculated using Cox proportional hazards models.<br>● All analyses will be conducted using Stata (version 17).

1. Research background

Gastric cancer (GC) is one of the most common malignancies worldwide[1], accounting for significant morbidity and mortality. Despite advancements in adjuvant therapies[2], surgery remains the main curative treatment for gastric cancer. In recent years, laparoscopic gastrectomy (LG) has been increasingly adopted as a minimally invasive alternative to open gastrectomy (OG) for advanced gastric cancer (AGC). Large-scale randomized controlled trials (RCT) such as CLASS-01[3] and KLASS-02[4] have established the non-inferiority of LG in terms of oncological outcomes compared to OG, with additional benefits such as reduced blood loss, shorter hospital stays, and faster postoperative recovery. These findings have led to the recommendation of LG as an alternative approach in treatment guidelines for locally AGC[2,5-7].

However, these studies have notable limitations, including a high proportion of stage I patients and a focus on T2–T3 tumors, resulting in limited representation of T4a cases. T4a tumors, characterized by serosal invasion, represent a particularly aggressive and challenging subset of gastric cancers. These tumors are often associated with large tumor sizes, extensive nodal metastases, and peritumoral inflammatory reactions, posing significant challenges to surgical management. Achieving an adequate D2 lymph node dissection laparoscopically in T4a cases is technically demanding, increasing the risk of intraoperative, postoperative complications and inadequate lymphadenectomy. Furthermore, T4a tumors carry a higher risk of recurrence, particularly peritoneal dissemination, and are associated with poorer prognoses. These risks may be exacerbated in laparoscopic procedures due to factors such as pneumoperitoneum and tumor manipulation, which could facilitate peritoneal seeding and trocar site metastasis[8,9].

Several retrospective studies had demonstrated the benefits of LG for T4a GC, including better surgical outcomes, such as operation time, blood loss, lower complication rates, shorter postoperative recovery, and comparable survival outcomes to OG[10-15]. However, the majority of these studies suffer from significant limitations, such as patient selection bias, which cannot be completely mitigated even with propensity score-matched method, small sample sizes, missing tumor’s characteristics, variation in surgical techniques and an

inadequate long-term follow-up data. The lack of high-quality evidence has limited the generalizability of these findings, and is insufficient to establish robust evidence on long-term oncological outcomes. Subgroup analysis of JLSSG0901 trial in patients with T4a disease suggested worse 5-year recurrence-free survival (RFS) in those underwent laparoscopic distal gastrectomy compared to open distal gastrectomy[16]. Although these findings highlight potential concerns, the trial was not specifically designed to evaluate outcomes in T4a cases, and definitive conclusions cannot be drawn. Currently, no RCTs have been conducted to directly compare LG and OG specifically for T4a gastric cancer. Thus, the efficacy of LG for T4a gastric cancer remains controversial, particularly regarding long-term survival outcomes. Given these gaps in the evidence, there is an urgent need for high-quality research to clarify the role of LG in the management of this high-risk GC subgroup. The rationale is to determine whether laparoscopic surgery provides equivalent oncological outcomes while reducing complications and enhancing recovery for patients with T4a GC. This RCT is conducted to compare laparoscopic and open distal gastrectomy with D2 lymphadenectomy in term of early outcomes and long-term survival for patients with resectable T4a GC.

2. Objective

Primary Objective:

- To assess whether LDG is non-inferior to ODG in terms of 3-year disease-free survival (DFS) in patients with cT4aN0-3M0 gastric cancer.

Secondary Objectives:

- To compare operative safety, surgical morbidity and mortality.
- To assess recovery indices: length of postoperative hospital stay, time to first flatus, time to oral tolerance, and time from surgery to initiation of adjuvant therapy.
- To assess quality of life (QoL) and late complications.

3. Research design

3.1 Study Design

This study is a single center, phase III, open-label, randomized controlled trial (RCT) with a parallel-group, non-inferiority design from University Medical Center Ho Chi Minh city, a tertiary medical center.

Participants are randomized 1:1 to receive LDG or ODG.

The primary outcome was 3-year disease free survival (DFS).

This RCT is monitored by an independent data and safety monitoring committee (DSMC) organized by the Department of Scientific Research and Training of Ho Chi Minh City University Medical Center.

Gastrop-intestinal Surgery Department, University Medical Center Ho Chi Minh city

3.2 Case group

Group A (study group): laparoscopic distal gastrectomy + D2 lymphadenectomy group (LDG group)

Group B (control group): Open distal gastrectomy + D2 lymphadenectomy group (ODG group)

3.3 Estimate Sample Size

To estimate the sample size for a non-inferiority RCT with 3-year DFS primary enpoint, the calculation is performed using the log-rank test for survival analysis in non-inferiority trials, with the use of a web-based tool developed by Kengo Nagashima, Keio University, Japan[17,18]. In a subgroup analysis for T4a GC, the estimation of 3-year DFS rates were 57% for the LDG group and 55% for the ODG group[15,19]. Therefore, a hazard ratio (HR) of 1.45 was used for the non-inferiority margin (Δ_0). The null hypothesis was assigned as HR ≥ HR0 , and the alternative hypothesis as HR < HR0 . Type I error was set at 0.05 (one-sided) with 80% power, with a 1:1 allocation ratio. The estimated total sample size required is 240 patients (120 patients in each group), with 116 target events as recurrence. This ensures that at least 206 patients (103 per group) will be analyzed in the intention-to-treat (ITT) population after considering for the 14% dropout rate. (**Figure 5**).

The estimated patient recruitment period for this study is approximately 60 months, ,

assuming a steady accrual rate, to complete the trial enrollment.

3.4 Randomization and blind method:

Patients are randomly assigned in a 1:1 ratio to the LDG or ODG group using block randomization with random block sizes of 2, 4, or 6, generated by Stata software (version 16). Allocation concealment is ensured through sealed envelopes. Due to the nature of surgical interventions, blinding of surgeons and patients is not possible. However, outcome assessors and data analysts remain blinded to group assignments.

4.Study objects

4.1 Inclusion criteria

- Age 18–80 years.
- ECOG performance status of 0 or 1.
- ASA score of I–III.
- Clinical diagnosis with T4aN0-3M0 gastric adenocarcinoma suitable for curative resection by distal gastrectomy with D2 lymphadenectomy based on preoperative imaging.
- Willingness to participate the study and written informed consent.

4.2 Exclusion criteria

- Bulky LNs on preoperative findings.
- Previous gastric surgery
- Severe tumor-related complications such as bleeding or perforation.
- Prior chemotherapy or radiotherapy.
- Diagnosis of other malignancies within the past 5 years.
- Severe comorbidities or vulnerable conditions (eg, cognitive impairment, ongoing or planned pregnancy) contraindicating laparoscopy

- Participation in another clinical trial

4.3 Withdrawal criteria

- M1 tumor confirmed intraoperatively and did not received gastrectomy aimed to curative treatment.
- Patients intraoperatively confirmed as T4b and did not received gastrectomy
- Sudden severe complications during the preoperative period (intolerable surgery or anesthesia), which renders it unsuitable or unfeasible to implement the study treatment protocol as scheduled;
- Patients confirmed to require emergency surgery due to changes in the patient’s condition after inclusion in this study;
- Patients who voluntarily quit or discontinue treatment for personal reasons at any stage after inclusion in this study;

4.4 Case screening

Patients will be recruited from the outpatient clinics and surgical departments of participating hospital. All patients undergo gastroscopy with histological confirmation prior to enrollment to ensure the diagnosis of gastric adenocarcinoma. Patients with gastric adenocarcinoma accessing the Gastro-Intestinal Surgery Department will be performed image staging with chest-abdomen Computed Tomography (CT), and Positron Emission Tomography if necessary. Perioperative assessment will be performed by a multidisciplinary team. An Independent Tumor Board (ITB) will be conducted to identify clinical staging and a decisive treatment plan. Patients who are determined as T4aGC to undergo distal gastrectomy with curative intention are potential subjects for this trial.

5. Diagnostic criteria for this study

All patients undergo gastroscopy with histological confirmation prior to enrollment to ensure the diagnosis of gastric adenocarcinoma. For the protocol of abdominal CT-scan, all patients received 500 mL water as an oral contrast agent approximately 15 min before the examination. The clinical T4a tumors are diagnosed based on one or more the following criterias on CT-Scan: (1) nodular or an irregular outer layer of the gastric wall, (2) haziness/ stranding of the perigastric fat, and (3) a hyperattenuating serosa sign.[20]

The diagnosis of cT4a stage based on CT scan findings will be determined by a team of consultant radiologists specialized in abdominal imaging (at least two experienced radiologists). All CT scans are interpreted independently and prospectively in the radiology department, with radiologists blinded to prior knowledge of treatment plans, to ensure objectivity and standardization. Then, a multidisciplinary team meeting will be conducted to determine the final clinical staging and treatment plan.

6. Outcome Measures

6.1 Primary Outcome Measures

The primary outcomes of this RCT are 3-year disease-free survival (DFS). To assess the 3-year DFS rate, clear criteria for recurrence are defined.

6.2 Secondary Outcome Measures

3-year overall survival (OS)

Surgical outcomes: Operating time, Blood loss, intra-operative bleeding > 200ml, blood transfusion, intra-operative organ injury, conversion to open surgery, R1 resection rate, number of retrieved LNs

Early complications: wound infections, intra-abdominal or intraluminal bleeding, fluid collection or abscess, anastmosis or duodenal stump leakage, anastomotic stenosis, pancreatic fistula, postoperative ileus, cardio-pulmonary complications

Postoperative recovery: Postoperative hospital stay, Time to flatus, Time to oral tolerance, Time interval between surgery and adjuvant chemotherapy

Late complications: intestinal obstruction related to adhesion, abdominal incision hernia, chronic wound infection

Quality of life

7. Qualifications of the participated Surgeons

7.1 Basic principle

All patients enrolled in this trial are performed only by members of upperGI surgical team. Prior to the initiation of this study, all participating surgeons had standardized their techniques for both open and laparoscopic gastrectomy with D2 lymphadenectomy for advanced gastric cancer. Each surgeon had independently performed at least 100 laparoscopic and 100 open gastrectomy procedures. A checkpoint list of surgical steps was used to validate the procedure.

7.2 Checklist for determination of success about D2 lymphadenectomy

	Yes	No
1. Properly total omentectomy	□	□
2. Ligation of left gastroepiploic vessels at origin for dissection of station 4sb	□	□
3. Ligation of right gastroepiploic artery at origin	□	□
4.Ligation of infra-pyloric artery at origin and adequate lymphadenectomy of station 6 (station6a, 6v, 6i)	□	□

5. Ligation of right gastric artery at origin (station 5)	□	□
6. Adequate and en-bloc lymphadenectomy of suprapancreatic area. (stations 8a, 9)	□	□
7. Adherence to the principle of the "outermost layer-oriented medial approach" [21] and "Pancreas-Compressionless"[22] methods	□	□
8. Exposure from right border of proper hepatic artery to left border of portal vein for dissection of station 12a	□	□
9. Ligation of left gastric vessels separately at origin (station 7)	□	□
10. Identification and dissection along splenic vein to posterior gastric artery for dissection of station 11p	□	□
11. Exposure of gastroesophageal junction for dissection of station 1 and 3.	□	□

8. End point and definition of related result determination

8.1 Disease-free survival (DFS)

Definition of recurrence:

- For patients without specific symptoms, recurrences are detected during regular follow-up investigations, such as abdominopelvic computed tomography (CT). If any suspicious findings are noted, further diagnostic procedures, including whole-body positron emission tomography-CT (PET-CT), magnetic resonance imaging (MRI) of the liver, or laparoscopic exploration, will be performed to confirm recurrence. In these cases, follow-up intervals are shortened, and patients are monitored more frequently than the standard schedule. For patients presenting with suspected symptoms of recurrence, evaluation for recurrence will be conducted immediately, irrespective of the planned follow-up schedule

- Definition of recurrence date:

(1) Recurrence is identified by imaging examinations (X-ray, ultrasound, CT, MRI, PET-CT, endoscope) without contradictory results. The earliest detection date is defined as the "recurrence date."

(2) If there is no use of imaging or a pathological diagnosis, the date of clinical recurrence based on clinical history and physical examination is defined as the “recurrence date”.

(3) For cases without imaging or clinical diagnosis but with a tissue biopsy pathological diagnosis of recurrence, the earliest date confirmed by biopsy pathology is considered the “recurrence date”.

8.2 Overall survival (OS)

Overall survival is determined from the date of surgery until death or the final follow-up date, whichever occurs first. For patients who survive, the endpoint is the last confirmed survival date. In cases where follow-up is lost, the endpoint is the last date that survival could be verified.

8.3 Surgical outcomes

Names of surgical outcomes	Definitions
Operating Time	Time in minutes from the first skin incision to the final skin closure. Measured in minutes
Estimated Blood Loss	Total intraoperative blood loss, estimated by suction volume and surgical sponges. Measured in minutes milliliters (mL)
Intraoperative Bleeding >200 mL	A binary variable (yes/no) indicating whether total blood loss exceeded 200 mL during surgery
Blood Transfusion	Administration of any allogeneic red blood cell product during or immediately after surgery.
Intraoperative Organ Injury	Any unintended injury to adjacent organs (e.g., liver, pancreas, spleen, bowel, vessels…) requiring repair or altering the surgical plan
Conversion to Open Surgery (for LDG group)	Any case initially planned as laparoscopic that required conversion to open laparotomy before completing D2 lymphadenectomy.
R1 Resection Rate	The proportion of patients with microscopic residual tumor (positive margins) at the proximal, distal, or radial margin on final pathology.<br>Or the introparative cytology is positive

Number of Retrieved Lymph Nodes	Total number of lymph nodes dissected and pathologically examined, indicating the extent of lymphadenectomy.

8.4 Recovery outcomes

Recovery outcomes will be assessed using standardized parameters reflecting the postoperative course. Data will be collected prospectively from the day of surgery until discharge, and include the following:

Names of recovery outcomes	Definitions
Time to first flatus (day)	Days from surgery to first passage of gas per rectum. Recorded based on patient’s self-report or nurse observation
Time to oral tolerance (days)	Days from surgery to first successful tolerance of oral fluids or soft diet.
Time from surgery to initiation of adjuvant chemotherapy (days)	Defined as the interval from the day of surgery to the first cycle of adjuvant chemotherapy in patients indicated for postoperative systemic treatment.
Postoperative hospital stay (days)	Defined from the date of operation to the date of hospital discharge
Readmission within 30 days	All-cause readmissions within 30 days post-discharge are recorded

Temperature will be monitored at least three times daily, and the maximum postoperative temperature within the first seven days will be documented as a surrogate marker of inflammatory response.

These parameters serve to evaluate the pace and quality of postoperative recovery, which is one of the secondary endpoints of the trial. Data from both groups will be compared to assess whether laparoscopic gastrectomy improves recovery compared to open surgery in the setting of T4a gastric cancer.

8.5 Postoperative complications

Complications were diagnosed and recorded based on the “International consensus on a complications list after gastrectomy for cancer[23].

Early complication: occurring within 30 days after surgery, will be classified using the Clavien-Dindo classification system[24], and divided into 2 subclassifications: (1) surgical

complications , (2) general complications

Classification and name of complication	Diagnostic criteria
**Surgical complications**	
Abdominal bleeding	Intra-abdominal hemorrhage requires blood transfusion, emergency endoscopy or surgical intervention to eliminate anastomotic bleeding
Anastomotic bleeding	The postoperative hematemesis or melena, or gastrointestinal tube continued to have fresh red blood outflow; the hemoglobin drops more than 1g/dL
Anastomotic leak	Full thickness defect of gastro-jejunal, jejuno-jejunal anastomoses irrespective of (i) presentation, (ii) method of identification, (iii) clinical consequences, and (iv) treatment. An abscess close to the anastomosis should also be recorded in this group. Detection of anastomotic leakage is achieved via gastrointestinal contrast radiography, demonstrating extravasation of contrast medium from the anastomotic site, or by observing methylene blue dye output through surgical drains following oral administration.
Duodenal stump leak	Definition: Full thickness duodenal defect irrespective of (i) presentation, (ii) method of identification, (iii) clinical consequences, and (iv) treatment. An abscess close to the duodenal stump should also be recorded in this group.
Anastomotic stricture	Mechanical obstruction of gastro-jejunal anastomosis, and endoscopic scope can not passing through the anastomosis.
Pancreatic fistula	A drain output of any measurable volume of fluid with an amylase level > 3 times the upper limit of institutional normal serum amylase activity, associated with a clinically relevant development/condition related directly to the postoperative pancreatic fistula (2016 International Study Group of Pancreatic Fistula's definition). A biochemical leak is not recorded as pancreatic fistula
Paralytic ileus	Presence of symptoms, such as abdominal distension, nausea, vomiting and markedly reduced or absent bowel sounds occurring after postoperative day 5, requiring refasting

	without mechanical obstruction.
Pancreatitis	is diagnosed if two conditions are met: (i) there exists a postoperative increase in serum amylases/lipases more than 3 times the normal value, and (ii) there are radiological signs of postoperative pancreatitis (e.g. edema or necrosis at CT scan)
Chyle leak	The drainage fluid is milky white, and more than 200ml/d and and does not decrease for 48 hour, and the level of triglyceride >110 mg/dL at the same time.
Intestinal obstruction after operation	Defined by : (i) clinical and radiological signs (abdominal X-ray or CT-Scan) of mechanical obstruction (ii) a patient's inability to enteral feed occurs, and (iii) there exists the need for nasogastric replacement beyond the normal postoperative course
Early dumping syndrome	The symptoms of sweating, heat, weakness, dizziness, palpitations, a feeling of heart swelling, vomiting, abdominal colic, or diarrhea combined with the signs of tachycardia, a slight increase in blood pressure, and slightly faster breathing occurring 15-30 minutes after a meal are indicative of rapid gastric emptying as observed through solid phase radionuclide gastric emptying scanning.
Late dumping syndrome	Feeling hungry, flustered, out of sweating 2-3 hours after the meal . Blood sugar is less than 2.9mmol/L, excluding other diseases that cause hypoglycemia
Internal hernia	Postoperative CT findings of cystic or cystic and solid mass, and intestinal aggregation, stretching, translocation, abnormal mesenteric movement, and thickening of the blood vessel.
Abdominal wound dehiscence	Including partial dehiscence of the incision and full-layer dehiscence
Wound infection	Presence thickening, swelling or pus at the incision, anh/or culture of wound secretions yielding pathogenic organisms.
Intra-abdominal abscess	Postoperative abnormal fluid abdominal collections without gastrointestinal leak(s) preventing drainage removal and/or requiring treatment
**General complications**	
Pneumonia	Complies with one of the following two diagnostic criteria: 1.

	Auscultation/percussion voiced + one of the following: fresh sputum or sputum character changes; blood culture (+); bronchoalveolar lavage fluid, anti-pollution sample brush, biopsy specimens cultured pathogenic bacteria. 2. Chest film hints of new or progressive infiltration + one of the following: fresh sputum or sputum character changes, blood culture (+), bronchoalveolar lavage fluid, anti-pollution sample brush, biopsy specimens cultured pathogenic bacteria; isolate virus or detect IgM, IgG (+) of respiratory viral
Pleural effusion/infection	CT scan showed the localized fluid low density area of thoracic cavity, which could accompany with gas, and culture pathogenic bacteria in thoracic endocrine.
Gastrointestinal infection	There is at least one of the following types of evidence in abdominal cavity within 30 days after operation: 1. discharge of pus, with/without microbiological examination; 2. bacterial culture positive; 3. diagnosed by detection, pathology, imaging findings.
Urinary system infection	Symptoms of urine frequency, urgency and urine pain etc. and urine bacteria culture colony count 1000~10 million/ml in the absence of antibiotics; No symptoms of urine frequency, urgency and urine pain etc, urine bacterial culture colony count ≥ 100,000/ml
Sepsis	The following two conditions are available: 1. There is evidence of active bacterial infection, but the blood culture does not necessarily appear pathogenic bacteria; 2. meeting two of the following four items at the same time: (1). body temperature >39. 0℃ or <35.5 ℃ for 3 consecutive days, (2). heart rate > 120 times/min; (3). total white blood cells >12. 0×10^9/L or <4.0×10^9/l, wherein neutrophils >0. 80, or naive granular cells >0. 10; (4). Respiratory frequency > 28 times/min
Cardiac dysfunction	The symptom of snus tachycardia, sinus bradycardia, supraventricular tachycardia, ventricular tachycardia, and other arrhythmias, or heart failure preoperatively none-existing and postoperatively appearing, and other causes of the above-mentioned manifestations are excluded.
Hepatic dysfunction	Bilirubin increasing and the levels of AST and ALT >5 times

	after operation and these symptoms no existing before surgery.
Kidney function failure	Postoperative continuing renal function insufficiency, blood creatinine rising 2mg/dl, or acute renal failure needing dialysis treatment.
Cerebral embolism	Acute onset, hemiplegia, aphasia and other focal neurological function deficits. Embolism site has low-density infarction, of which border is not clear and no obstruction performance within 24-48 hours after the onset.
Pulmonary embolism	Characteristics of dyspnea, chest pain, syncope, shortness of breath, right ventricular insufficiency and hypotension, pulmonary angiography revealed a filling defect.
Venous thrombosis of lower extremities	Local tenderness, swelling, purple skin color, combined with intravenous angiography to show the filling defect
Other	Complications other than the above complications, which do not exist before surgery but appear after surgery

Late complications: defined as those occurring after 30 days

Classification and name of complication	Diagnostic criteria
Incisional hernia of abdominal wall	The swelling tumor showing in the surgical scar area or abdominal wall swelling when standing or force. CT shows ventral wall continuity interruption and hernia content extravasation
Chronic wound infection	Thickening of the soft tissue at the incision, in or below the incision of gas, exudation, swelling of the incision or pus from the incision extrusion, or secretion culture of pathogenic bacteria.
Intestinal obstruction related to adhesion	Abdominal X-ray shows a plurality of liquid planes and the phenomenon of intestinal effusion with visible isolated, fixed, swelling of the intestinal loop. Total Abdominal CT showed edema, thickening, adhesion of intestinal wall, accumulation of gas in intestinal cavity, uniform expansion of bowel and intra-abdominal exudation.

8.6 Mortality

Overall Mortality

Defined as the proportion of deaths from any cause within the study population.

Cancer-Specific Mortality

Refers to deaths directly attributable to the progression or recurrence of gastric cancer. Determined based on clinical records, death certificates, or multidisciplinary review.

Postoperative Mortality

Defined as death occurring within 30 days following surgery or during the same hospital admission.

9. Standard trial proceedings

9.1 Patient selection

- Patients will be recruited from the outpatient clinics and surgical departments of participating hospital. All patients undergo gastroscopy with histological confirmation prior to enrollment to ensure the diagnosis of gastric adenocarcinoma. Patients with gastric adenocarcinoma accessing the Gastro-Intestinal Surgery Department will be performed image staging with chest-abdomen Computed Tomography (CT), and Positron Emission Tomography if necessary. Perioperative assessment will be performed by a multidisciplinary team. An Independent Tumor Board (ITB) will be conducted to identify clinical staging and a decisive treatment plan.

- Patients who are determined as T4aGC to undergo distal gastrectomy with curative intention are potential subjects for this trial.

- For the protocol of abdominal CT-scan, all patients received 500 mL water as an oral

contrast agent approximately 15 min before the examination. The clinical T4a tumors are diagnosed based on one or more the following criterias on CT-Scan: (1) nodular or an irregular outer layer of the gastric wall, (2) haziness/ stranding of the perigastric fat, and (3) a hyperattenuating serosa sign.[20]

- The diagnosis of cT4a stage based on CT scan findings will be determined by a team of consultant radiologists specialized in abdominal imaging (at least two experienced radiologists). All CT scans are interpreted independently and prospectively in the radiology department, with radiologists blinded to prior knowledge of treatment plans, to ensure objectivity and standardization. Then, a multidisciplinary team meeting will be conducted to determine the final clinical staging and treatment plan.

- Following consent, patients will be randomized into either the LDG or ODG group. Patients will undergo surgery within 30 days of randomization. Perioperative care and follow-up adhere to standard guidelines for GC management.

9.2 Procedures

Intervention Arms:

A: Laparoscopic distal gastrectomy procedure

Prophylactic antibiotics are used routinely

Step 1: Trocar Placement and Comprehensive Assessment

During abdominal cavity assessment, cases with peritoneal metastasis, tumors invading surrounding organs, or requiring total gastrectomy are excluded from this RCT. For

resectable distal gastrectomy cases, peritoneal lavage is conducted at the subdiaphragmatic and Douglas areas using 250–300 mL of normal saline, collecting at least 200 mL for cytology (**Figure 1**).

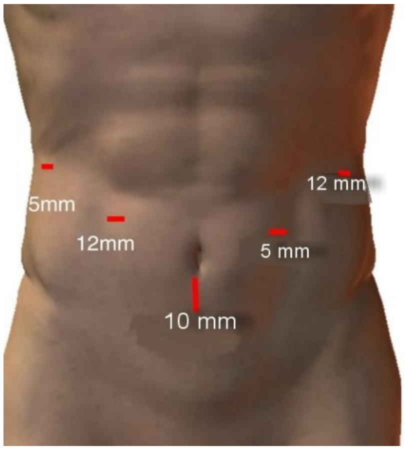

Figure 1 Trocar placements

Step 2: Dissection of the Greater Omentum and Lymph Node Stations 4sb and 4d

Total omentectomy was performed by dividing the greater omentum along the transverse mesocolon. The LGEA and LGEV are ligated at their origins from the splenic artery and vein, and adipose tissue containing station 4sb lymph nodes is dissected. The short gastric arteries are preserved during this process. Dissection continues along the greater curvature of the stomach to remove station 4d lymph nodes(**Figure 2**).

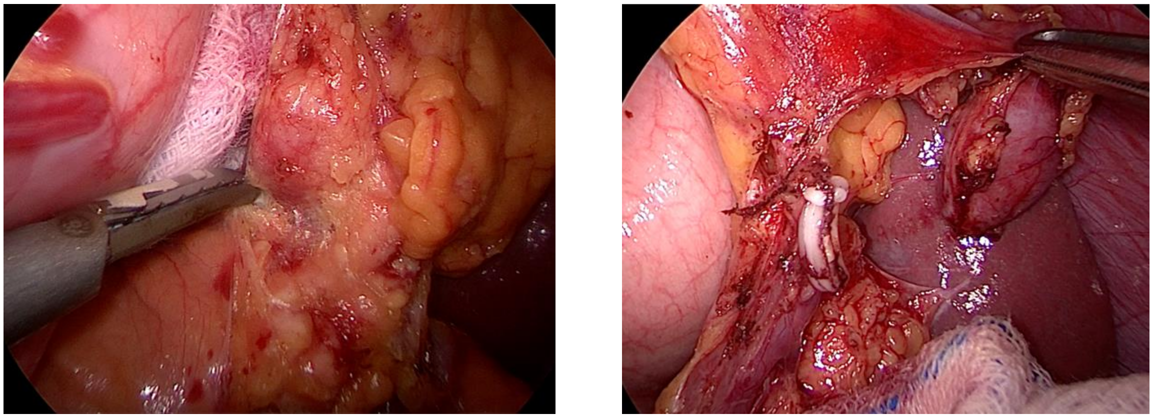

Figure 2 Dissection of group 4sb

Step 3: Dissection of Station 6

A plane between the omentum and transverse colon is dissected to expose the duodenum and pancreatic head. The RGEV is identified at its confluence with Henle's trunk, serving as the inferior boundary for station 6v. The RGEV is ligated, and adipose tissue containing station 6 nodes is carefully dissected off the pancreatic head, preserving the pancreatic capsule to avoid damage to the parenchyma. The RGEA is divided at its origin from the gastroduodenal artery, and infrapyloric vessels are ligated to complete the en bloc removal of station 6 lymph nodes (**Figure 3).**

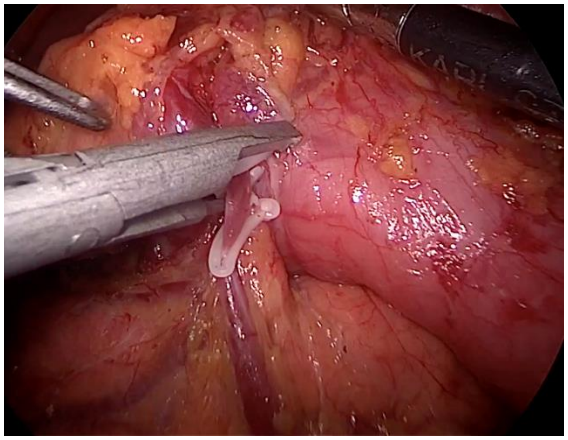

Ligated the RGEV

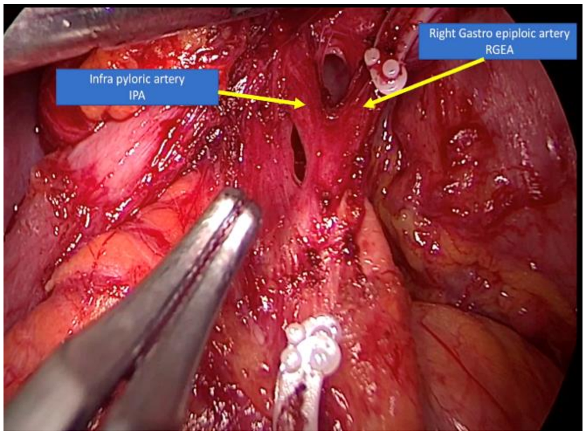

IPA and RGEA

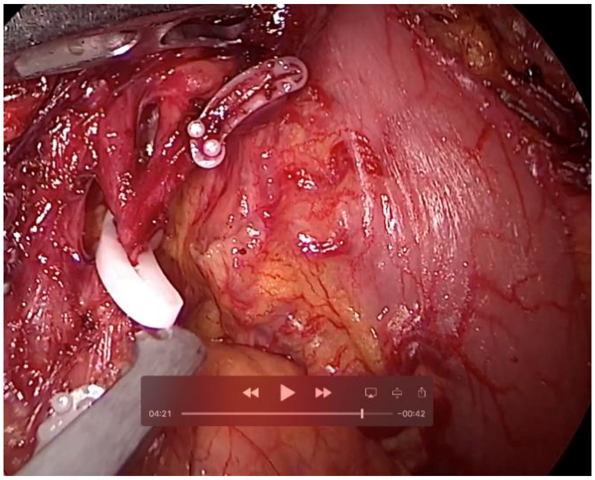

Ligate the IPA and RGEA together

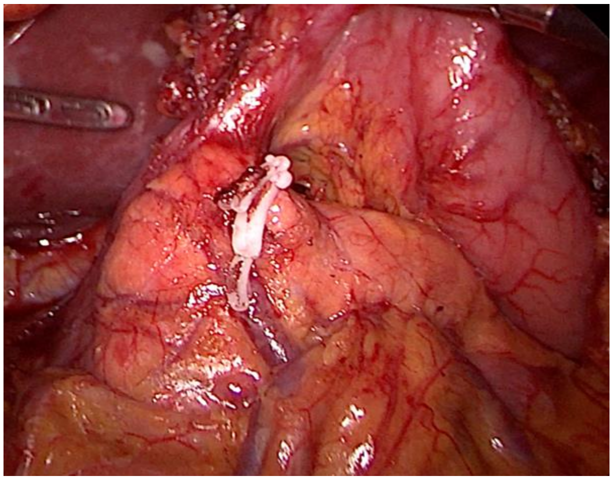

After LNs dissection group

Figure 3 Dissection of group 6

Step 4: Dissection of Stations 5 and 12a

The lesser omentum is divided along the left lobe of the liver to the cardia. The peritoneum above the duodenum is incised, and the lymphatic tissue of station 12a is dissected along the left side of common bile duct and down to the left side of the portal vein. The right gastric artery is ligated at its origin from the proper hepatic artery to retrieve stations 5 and 12a lymph nodes(**Figure 4**). The duodenum is transected 2 cm distal to the pylorus using a

linear stapler. After duodenal transection, the duodenal stump is routinely reinforced in all cases by inverting the stapler line using hand-sewn seromuscular sutures(**Figure 5**).

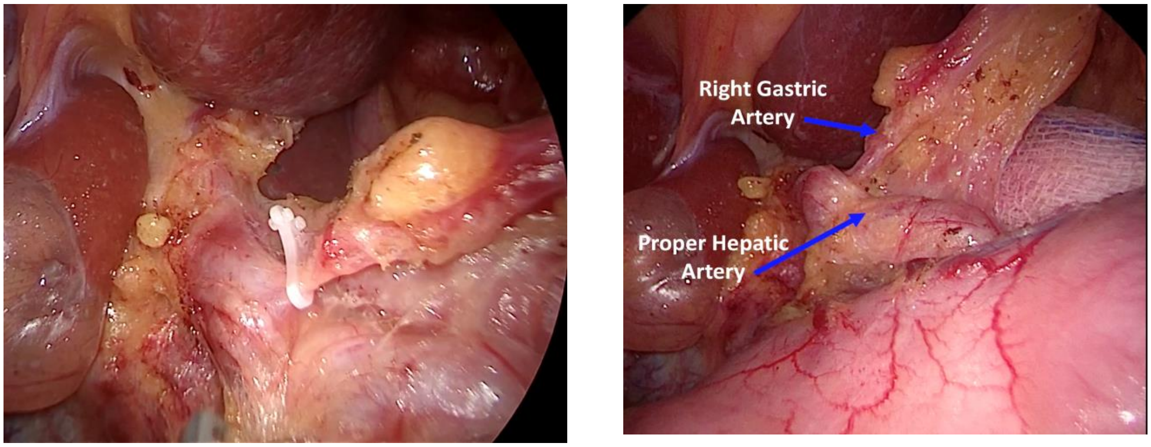

Ligate RGA for group 5

Lankmark of group 5

Figure 4 Dissection of group 5

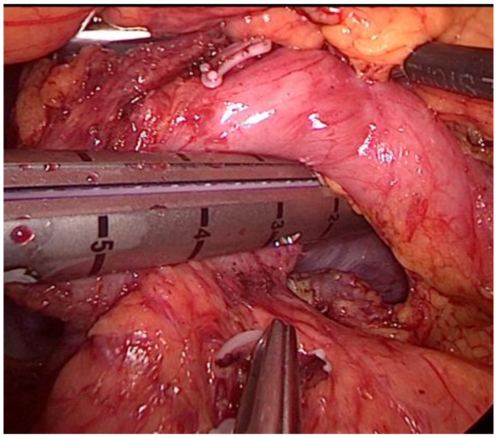

Figure 5 Duodenal transection

Step 5: Suprapancreatic Lymph Node Dissection (Stations 7, 8a, 9, and 11p)

Suprapancreatic lymph node dissection involves the removal of nodes in stations 7, 8a, 9, and 11p, located around major vessels such as the common hepatic artery, splenic artery, and celiac trunk. Using the “outermost layer-oriented medial approach” method [21,25,26], a surgical approach including (1) medial dissection of the left gastric artery; (2) right suprapancreatic dissection; (3) left suprapancreatic dissection was performed. The

outermost layer between the autonomic nerve sheaths and the lymphatic tissue is dissected. The avascular space of the left gastric artery (LGA) is dissected bilaterally and ligated the left gastric artery at its root for station 7. The adipose tissue containing station 8a is dissected along outermost layer of the common hepatic artery, dissection along the splenic artery for station 11p, and en bloc removal of tissue around the celiac trunk for station 9. (**Figure 6**)

We expose this area using a compressionless technique, including: (1) the assistant 's left hand uses laparoscopic forceps to grasp the fatty tissue at the inferior border of the pancreas, pulling it downward and posteriorly, (2) grasping and pulling the connective tissue surrounding the major blood vessels (outermost layer) (**Figure 7**) to expose and dissect along the superior border of the pancreas.

Dissection proceeds along the lesser curvature of the stomach to retrieve stations 1 and 3 lymph nodes **(Figure 8).**

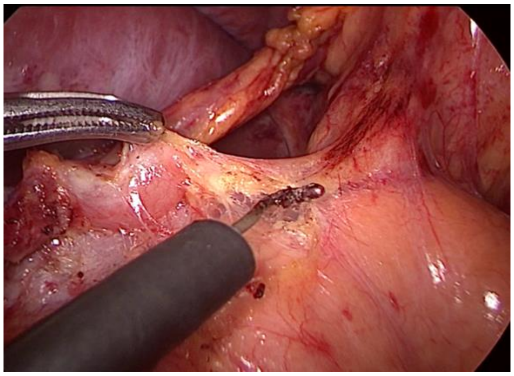

Open suprapacreatic border

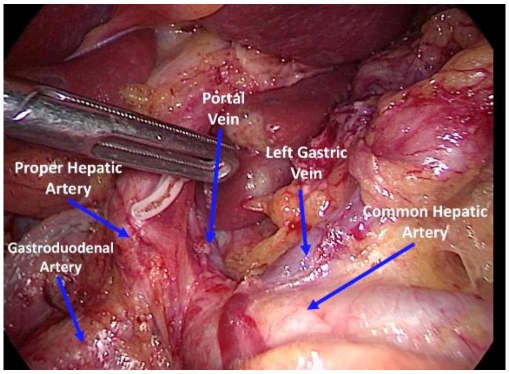

Lankmard for supra pacratic dissec

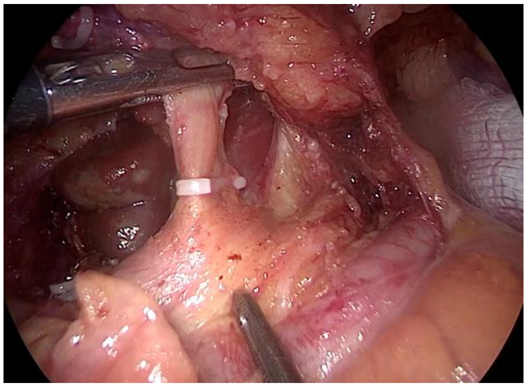

Ligate LGA for group 7

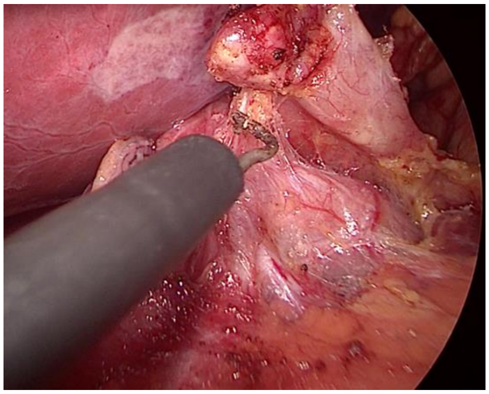

Outermost layer

Figure 6 Dissection of suprapancreatic are

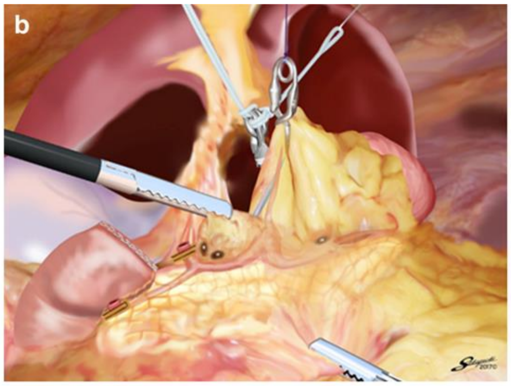

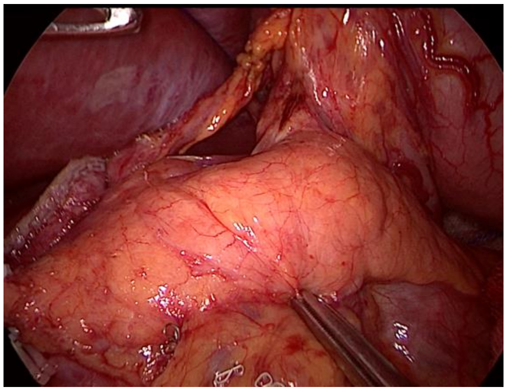

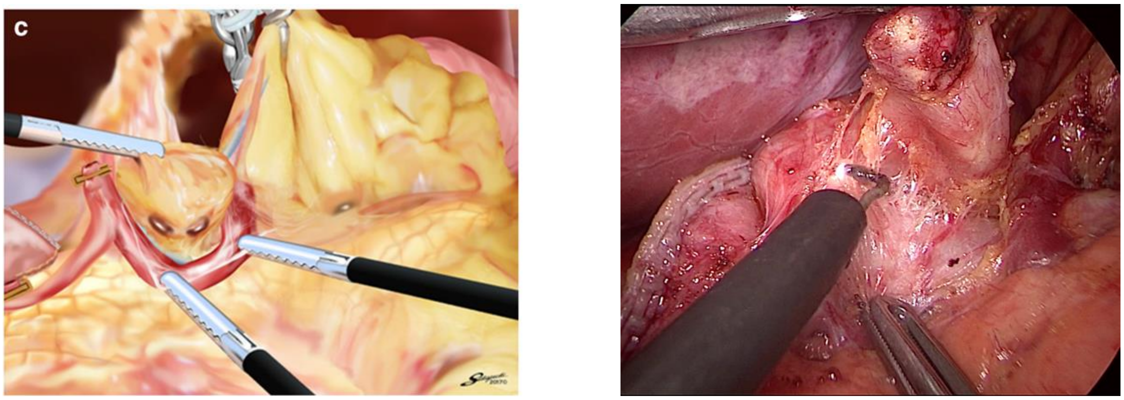

Figure 7 compressionless technique

Step 6: Lymph Node Dissection Along the Lesser Curvature (Stations 1 and 3)

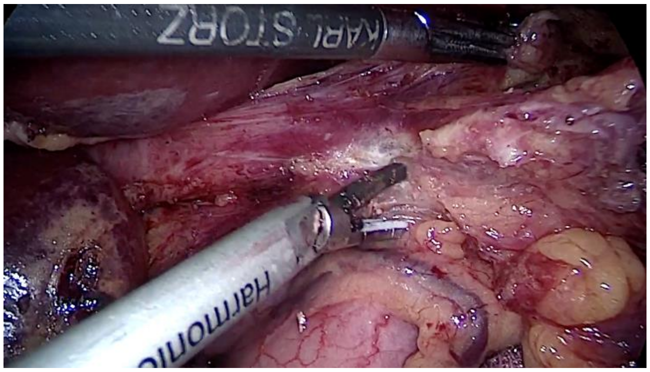

Figure 8 Dissection of group 1, 3

Step 7: Gastric Transection and Reconstruction

The stomach is transected at least 5 cm proximal to the tumor using a linear stapler. Specimens are extracted through a small abdominal incision, and examination of proximal margin is performed, including frozen resection if necessary. A post-gastrectomy cytology is performed in the surgical area routinely. Gastrointestinal reconstruction is performed using either Billroth II or Roux-en-Y methods. All Billroth II anastomoses are completed entirely intracorporeally. For Roux-en-Y reconstruction, the gastrojejunostomy are performed intracorporeally, while the jejunojejunostomy (Y-limb anastomosis) is constructed extracorporeally through the umbilical incision. A drainage tube is placed routinely **(Figure**

9).

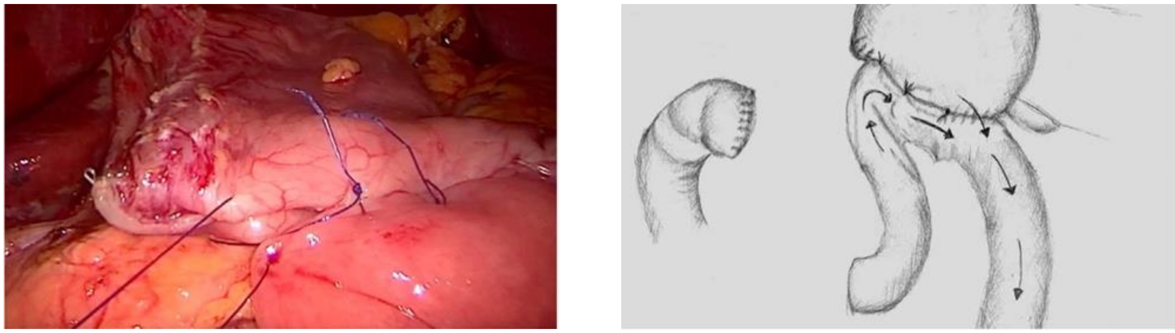

Figure 9 Modified Billroth II anastomosis

If a laparotomy is performed before finishing D2 lymphadenectomy for any reason, it will be recorded as “conversion to open”.

B: Conventional open distal gastrectomy procedure (Control arm)

The ODG procedure is similar to that of LDG in accordance with the instructions of the 6th Japanese Gastric Cancer Treatment Guidelines2.

All procedures included in this study are strictly performed by five senior consultant surgeons from our upper gastrointestinal surgical team. Each surgeon has personally conducted more than 100 standard open and laparoscopic gastrectomies with D2 lymphadenectomy for gastric cancer prior to the initiation of this study.

Specimen characteristics

Immediately after resection, the surgical specimen is extracted from the abdominal cavity and processed in accordance with a standardized protocol. All regional lymph nodes are dissected and grouped separately by anatomical stations based on the Japanese Gastric Cancer Association (JGCA) classification to allow accurate pathological correlation.

The stomach is then opened longitudinally along the side opposite to the tumor (most commonly along the greater curvature) to expose the tumor and both resection margins. Tumor size is measured along its largest dimension. The macroscopic type is classified according to the Borrmann classification system. The proximal and distal resection margins are measured and documented. If the tumor lies close to the resection margin, the surgeon may perform additional resection intraoperatively to achieve a safe margin, or a frozen section analysis may be conducted to confirm margin clearance **(Figure 10).**

High-resolution photographs of the fresh specimen are taken to document tumor location, macroscopic appearance, and resection margins (**Figure 11**). Special attention is paid to the serosal surface adjacent to the tumor. Gross serosal changes such as nodularity, loss of peritoneal shine, or whitish discoloration are recorded, as this trial focuses specifically on T4a gastric cancer.

The fresh specimen is then immediately delivered to the Department of Pathology. Histopathological evaluation includes tumor histological type and grade, depth of invasion, lymphovascular and perineural invasion, lymph node status (number retrieved and number positive), and resection margin status (R0 vs R1). Particular attention is also given to evaluating serosal involvement, as this trial specifically targets clinically diagnosed T4a gastric cancer.

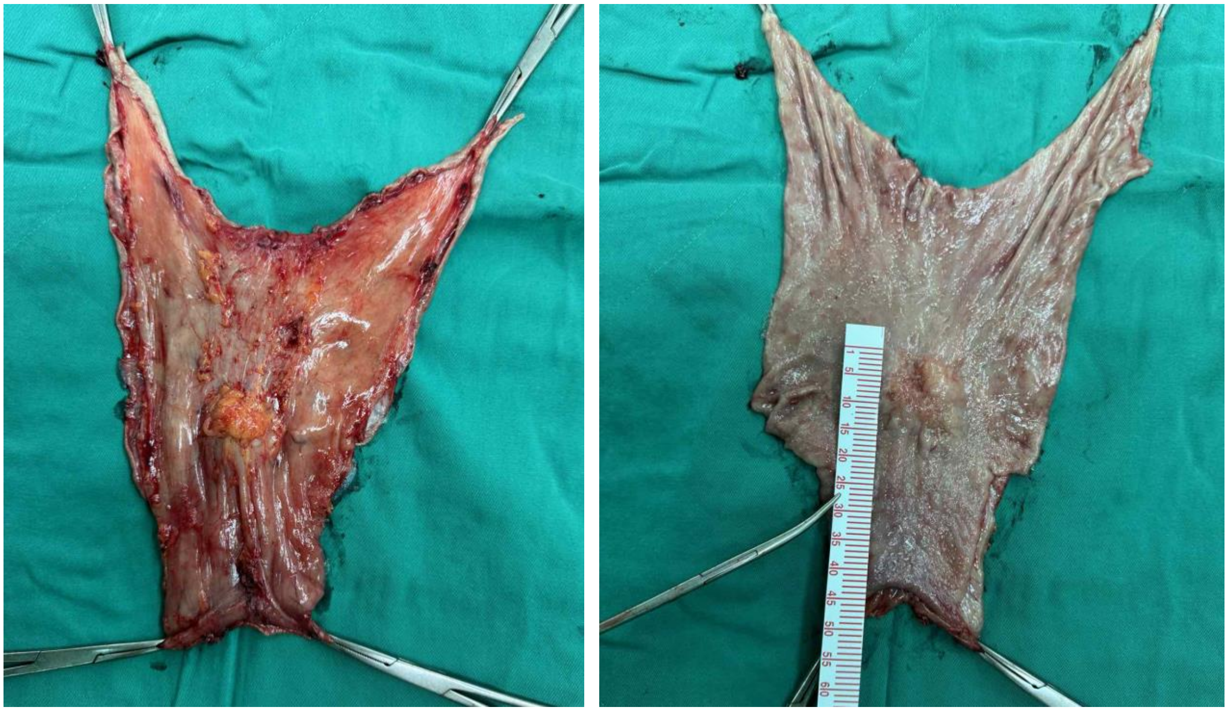

Figure 10 . Specimen: T4a tumor and tumor size measurement

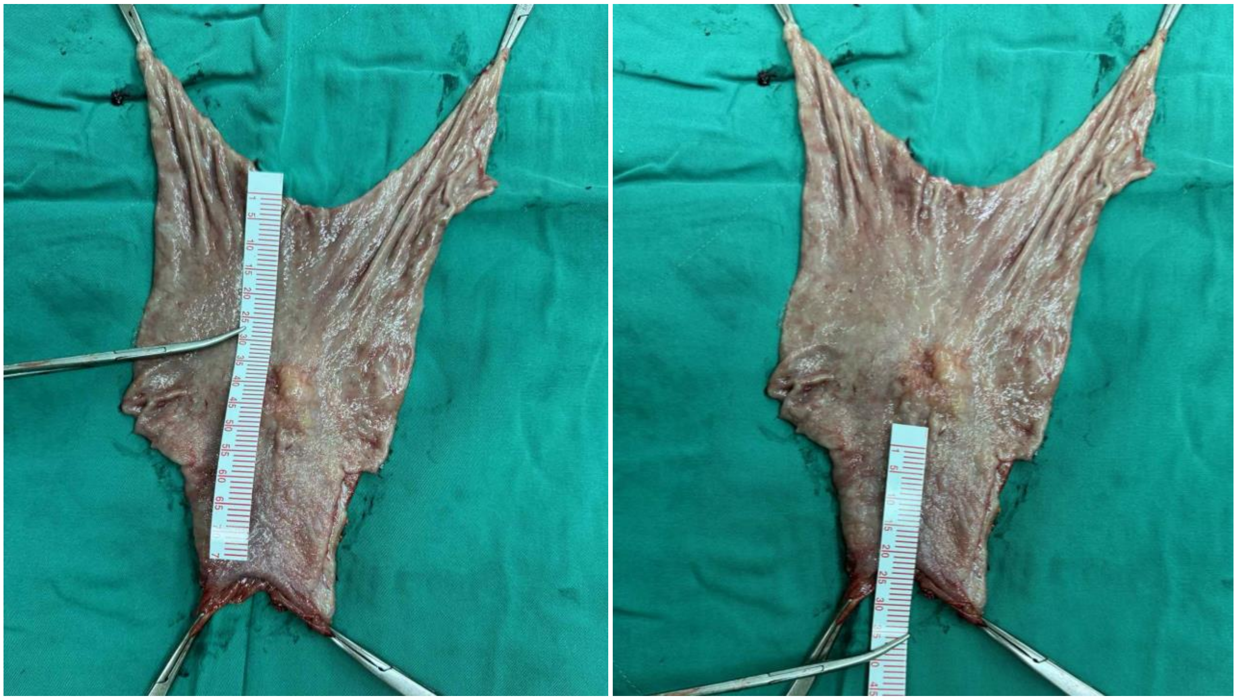

Specimen: Proximal margin measurement Specimen: Distalmargin measurement

Figure 11 Specimen: Proximal and distal margin measurement

Preoperative managements

All patients with surgical indications underwent routine screening for nutritional risk and thromboembolic risk. Those with a nutritional risk score (NRS) of 2 or higher received preoperative nutritional support for an average of 2–7 days, as recommended by a clinical nutrition specialist. Perioperative thromboprophylaxis was typically initiated preoperatively using mechanical measures such as early mobilization, physiotherapy, or compression stockings, particularly in patients with limited mobility and no evidence of lower extremity deep vein thrombosis on vascular ultrasound.

Patients also underwent preoperative respiratory physiotherapy as part of the Enhanced Recovery After Surgery (ERAS) protocol. For patients with underlying medical conditions, comorbidities were optimized and brought under control to an acceptable level prior to surgery.

Postoperative treatment and follow up

After surgery, all patients will be followed regularly using the same protocol, and relevant data, including recurrence and mortality, were recorded. Follow-up visits are scheduled every 3 months for the first 2 years postoperatively and every 6 months for the subsequent 3 years, ensuring a minimum follow-up period of 36 months for all patients. The follow-up protocol adheres to the 6th Edition of the Japanese Gastric Cancer Treatment Guidelines. **(Table 1)**

Duration after surgery	year					1				2	2.5	3	3.5	4	4.5	5
	month	1	3	6	9	12	15	18	21	24						
Medical examination, PS, Body weight		○	○	○	○	○	○	○	○	○	○	○	○	○	○	○
Blood test including tumor markers		○	○	○	○	○	○	○	○	○	○	○	○	○	○	○
CT and/or US				○		○		○		○	○	○		○		○
Endoscopy						○						○				○

Table 1 follow-up protocol

Post-operative adjuvant therapy

All patients undergoing surgery will be evaluated for postoperative adjuvant chemotherapy based on pathological staging, performance status, and recovery status, regardless of resection margin status.

At our center, adjuvant chemotherapy is routinely recommended for patients with pathological stage II or III disease, in accordance with current clinical guidelines. Chemotherapy is intended to begin within 4 to 6 weeks postoperatively, after the patient has adequately recovered and is deemed fit for systemic treatment, two standard regimens are used in clinical practice: capecitabine plus oxaliplatin (XELOX) and S-1-based chemotherapy (CS, DS, SOX, CapeOX), which may be administered either as monotherapy or in combination, depending on the patient’s condition and physician’s decision.

If a patient experiences severe side effects and is unable to tolerate the chemotherapy, the drug dosage should be reduced, or chemotherapy may be discontinued entirely. In this case, the patient is recorded as incomplete adjuvant chemotherapy.

The following variables will be recorded:

- Proportion of patients receiving adjuvant chemotherapy (%)

- Time from surgery to initiation of adjuvant therapy (days)
- Number of cycles completed
- Reasons for non-initiation or early discontinuation

These data will be included in secondary analyses to assess the feasibility and timing of postoperative chemotherapy following laparoscopic versus open distal gastrectomy for T4a gastric cancer.

10. Data management

Data management will be handled through a centralized electronic data capture (EDC) system. Trained personnel will perform data entry, ensuring accuracy through double-checking. Data will be securely stored with restricted access, and regular audits will maintain data quality.

An independent Data Safety Monitoring Committee (DSMC) will be assembled with 3 members from the Department of Scientific Research and Training of Ho Chi Minh City University Medical Center. An interim analysis will be performed after the enrolment of the first 120 participants. The DSMC will assess the following content:

- the number of participants and planned time for completion;
- drop rate of participants and compliance with procedures in each group;
- preliminary analysis of efficacy, including rate of complications and recurrence.
- incidence and classification of adverse events.

The interim analysis will be conducted using a two-sided significant test with the Haybittle–Peto spending function and a Type I error rate of 5% with stopping criteria of $P < 0.001$ (Z alpha = 3.29).

Independent study monitoring will be performed monthly by the Department of Scientific Research and Training of Ho Chi Minh City University Medical Center to ensure adherence to the protocol, International Conference on Harmonisation-Good Clinical Practice, standard operating procedures and applicable regulatory requirements, maintenance of trial-related source records, completeness, and accuracy and verifiability of case report form entries compared with source data.

11. Statistical Analysis

Outcomes will be analyzed using intention-to-treat (ITT), per-protocol (PP), and full analysis set (FAS) populations. Patients who crossover treatments preoperatively will be analyzed in the as-treated group (based on the actual procedure performed), while those converting from laparoscopic to open surgery intraoperatively remain in the laparoscopic group.

Summary statistics are mean ± standard deviation or median (interquartile range) for continuous variables and frequency and percentage for categorical variables. Continuous variables are compared using t-tests or Mann–Whitney U tests. Categorical variables ared analyzed using chi-squared or Fisher’s exact tests. Kaplan–Meier method is used to estimate OS and DFS, with comparisons between groups using the log-rank test. Hazard ratios with 95% is calculated using Cox proportional hazards models.

All analyses will be conducted using Stata (version 17).

12. Ethical approval

The study is conducted according to the guidelines of the Declaration of Helsinki and approved by Institutional Review Board, University Medical Center Ho Chi Minh city. Approval to perform research on human subjects in this study was provided by the Institutional Review Board, University Medical Center Ho Chi Minh city (registration number: 26/HDDD-DHYD) in June, 11th, 2020.

Informed consent will be obtained in writing from all patients prior to their enrollment in the study.

This trial was registered at ClinicalTrials.gov (NCT04384757). The study was first registered on May 08, 2020.

This RCT is monitored by an independent data and safety monitoring committee (DSMC) organized by the Department of Scientific Research and Training of Ho Chi Minh City University Medical Center.

The findings of this trial will be published in peer-reviewed journals and presented at international conferences. Additionally, data will be shared on ClinicalTrials.gov to meet transparency requirements. De-identified participant data will be accessible upon reasonable request following the trial's publication, in accordance with institutional and ethical guidelines.

REFERENCE

1. Bray F, Ferlay J, Soerjomataram I, Siegel RL, Torre LA, Jemal A. Global cancer statistics 2018: GLOBOCAN estimates of incidence and mortality worldwide for 36 cancers in 185 countries. *CA Cancer J Clin*. Nov 2018;68(6):394-424. doi:10.3322/caac.21492

2. Japanese Gastric Cancer Treatment Guidelines 2021 (6th edition). *Gastric Cancer*. Jan 2023;26(1):1-25. doi:10.1007/s10120-022-01331-8

3. Huang C, Liu H, Hu Y, et al. Laparoscopic vs Open Distal Gastrectomy for Locally Advanced Gastric Cancer: Five-Year Outcomes From the CLASS-01 Randomized Clinical Trial. *JAMA Surg*. Jan 1 2022;157(1):9-17. doi:10.1001/jamasurg.2021.5104

4. Hyung WJ, Yang HK, Park YK. Long-term outcomes of laparoscopic distal gastrectomy for locally advanced gastric cancer: the KLASS-02-RCT randomized clinical trial. *J Clin Oncol*. 2020// 2020;38doi:10.1200/JCO.20.01210

5. Kim TH, Kim IH, Kang SJ, et al. Korean Practice Guidelines for Gastric Cancer 2022: An Evidence-based, Multidisciplinary Approach. *J Gastric Cancer*. Jan 2023;23(1):3-106. doi:10.5230/jgc.2023.23.e11

6. Ajani JA, D'Amico TA, Bentrem DJ, et al. Gastric Cancer, Version 2.2022, NCCN Clinical Practice Guidelines in Oncology. *J Natl Compr Canc Netw*. Feb 2022;20(2):167-192. doi:10.6004/jnccn.2022.0008

7. Lordick F, Carneiro F, Cascinu S, et al. Gastric cancer: ESMO Clinical Practice Guideline for diagnosis, treatment and follow-up. *Ann Oncol*. Oct 2022;33(10):1005-1020. doi:10.1016/j.annonc.2022.07.004

8. Ikeguchi M, Oka A, Tsujitani S, Maeta M, Kaibara N. Relationship between area of serosal invasion and intraperitoneal free cancer cells in patients with gastric cancer. *Anticancer Res*. Sep-Oct 1994;14(5b):2131-4.

9. Du J, Yu PW, Tang B. Application of stereology to study the effects of pneumoperitoneum on peritoneum. *Surg Endosc*. Feb 2011;25(2):619-27. doi:10.1007/s00464-010-1235-3

10. Kanaji S, Urakawa N, Mukoyama T, et al. Safety of Laparoscopic Gastrectomy for Advanced Gastric Cancer With Greater than Serosal Invasion Depth. *Anticancer Res*. Jul 2023;43(7):3145-3152. doi:10.21873/anticanres.16487

11. Zhang L, Zang L, Sun J, et al. Long-term Outcomes of Laparoscopy-assisted Gastrectomy for T4a Advanced Gastric Cancer: A Single-center Retrospective Study. *Surg Laparosc Endosc Percutan Tech*. Dec 2019;29(6):476-482. doi:10.1097/SLE.0000000000000684

12. Long VD, Dat TQ, Thong DQ, et al. Long-Term Outcomes of Open Versus Laparoscopic Distal Gastrectomy for T4a Gastric Cancer: A Propensity Score-Matched Cohort Study. *Ann Surg Oncol*. Apr 2023;30(4):2278-2289. doi:10.1245/s10434-022-12897-z

13. Long VD, Nguyen DT, Thong DQ, et al. Laparoscopic versus open total gastrectomy for T4a gastric cancer: a propensity score-matched analysis of long-term outcomes. *Updates Surg*. Jun 16 2024;doi:10.1007/s13304-024-01910-7

14. Pang H, Yan M, Zhao Z, et al. Laparoscopic versus open gastrectomy for nonmetastatic T4a gastric cancer: a meta-analysis of reconstructed individual participant data from propensity score-matched studies. *World J Surg Oncol*. May 29 2024;22(1):143. doi:10.1186/s12957-024-03422-5

15. Zhang F, Lan Y, Tang B, Hao Y, Shi Y, Yu P. Comparative study of laparoscopy-assisted and open radical gastrectomy for stage T4a gastric cancer. *Int J Surg*. May 2017;41:23-27. doi:10.1016/j.ijsu.2017.01.116

16. Etoh T, Ohyama T, Sakuramoto S. Five-year survival outcomes of laparoscopy-assisted vs open distal gastrectomy for advanced gastric cancer: the JLSSG0901 randomized clinical trial. *JAMA Surg*. 2023// 2023;158doi:10.1001/jamasurg.2023.0096

17. Jung S-H, Chow S-C. On Sample Size Calculation for Comparing Survival Curves Under General Hypothesis Testing. *Journal of Biopharmaceutical Statistics*. 2012/05/01 2012;22(3):485-495. doi:10.1080/10543406.2010.550701

18. K N. A sample size determination tool for the log-rank test of non-inferiority [Internet]. 20XX YYY ZZ. https://nshi.jp/en/js/twosurvyrni/.

19. Shi Y, Xu X, Zhao Y. Long-term oncologic outcomes of a randomized controlled trial comparing laparoscopic versus open gastrectomy with D2 lymph node dissection for advanced gastric cancer. *Surg*. 2019// 2019;165doi:10.1016/j.surg.2019.01.003

20. Kim TU, Kim S, Lee JW, Lee NK, Jeon TY, Park DY. MDCT features in the differentiation of T4a gastric cancer from less-advanced gastric cancer: significance of the hyperattenuating serosa sign. *Br J Radiol*. Sep 2013;86(1029):20130290. doi:10.1259/bjr.20130290

21. Uyama I, Suda K, Satoh S. Laparoscopic surgery for advanced gastric cancer: current status and future perspectives. *J Gastric Cancer*. Mar 2013;13(1):19-25. doi:10.5230/jgc.2013.13.1.19

22. Tsujiura M, Hiki N, Ohashi M, et al. "Pancreas-Compressionless Gastrectomy": A Novel Laparoscopic Approach for Suprapancreatic Lymph Node Dissection. *Ann Surg Oncol*. Oct 2017;24(11):3331-3337. doi:10.1245/s10434-017-5974-4

23. Baiocchi GL, Giacopuzzi S, Marrelli D, et al. International consensus on a complications list after gastrectomy for cancer. *Gastric Cancer*. Jan 2019;22(1):172-189. doi:10.1007/s10120-018-0839-5

24. Clavien PA, Barkun J, de Oliveira ML, et al. The Clavien-Dindo classification of surgical complications: five-year experience. *Ann Surg*. Aug 2009;250(2):187-96. doi:10.1097/SLA.0b013e3181b13ca2

25. Kanaya S, Haruta S, Kawamura Y, et al. Video: laparoscopy distinctive technique for suprapancreatic lymph node dissection: medial approach for laparoscopic gastric cancer surgery. *Surg Endosc*. Dec 2011;25(12):3928-9. doi:10.1007/s00464-011-1792-0

26. Uyama I, Kanaya S, Ishida Y, Inaba K, Suda K, Satoh S. Novel integrated robotic approach for suprapancreatic D2 nodal dissection for treating gastric cancer: technique and initial experience. *World J Surg*. Feb 2012;36(2):331-7. doi:10.1007/s00268-011-1352-8

Appendix 1: FLOW DIAGRAM FOR RCT LDG vs ODG

FLOW DIAGRAM FOR RCT LDG vs ODG

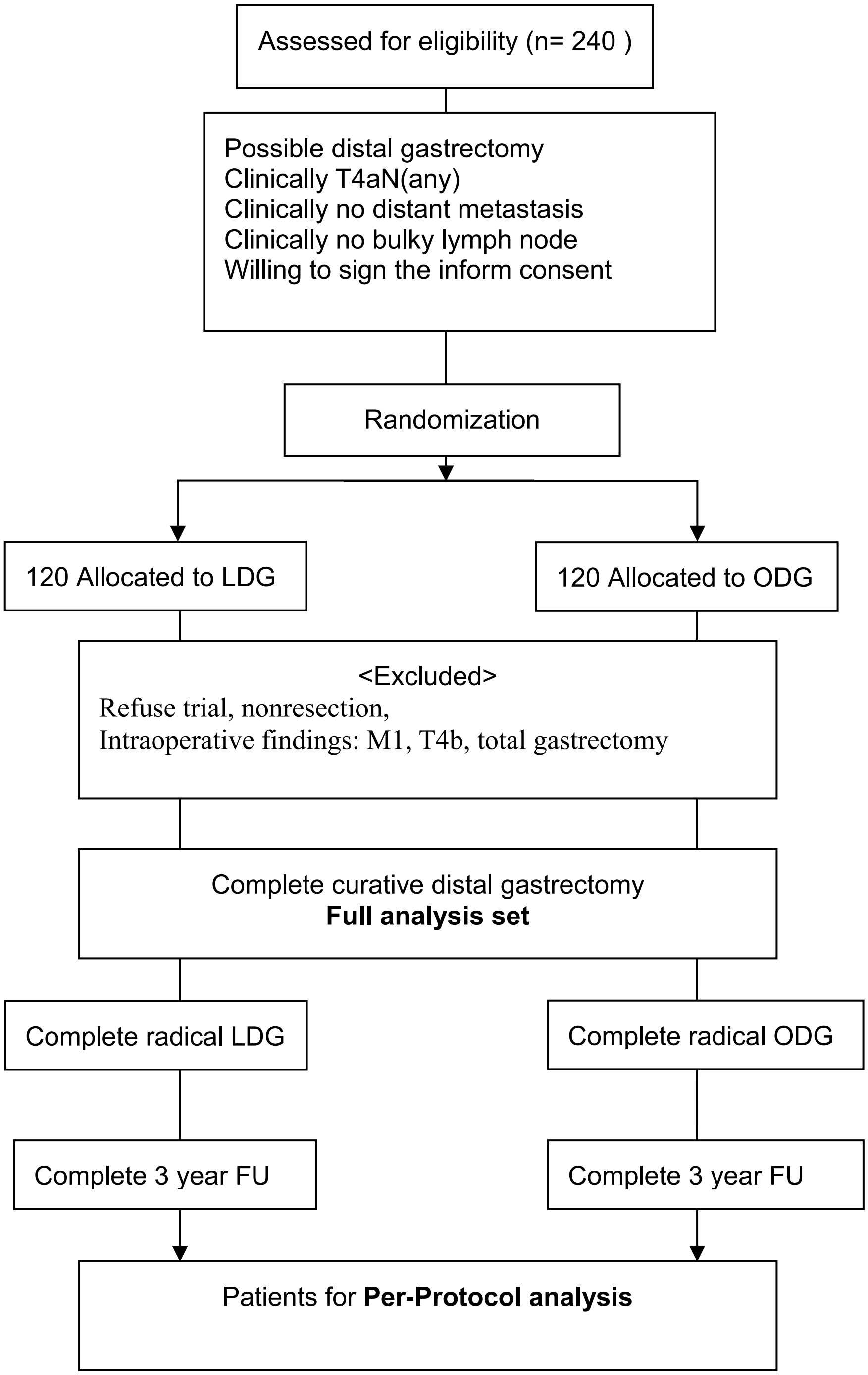

Appendix 2: INFORMED CONSENT FORM

INFORMED CONSENT FORM

Title of the Study: A Randomized Controlled Trial Comparing Laparoscopic and Open Distal Gastrectomy with D2 Lymphadenectomy in the Treatment of Locally T4a Gastric Cancer.

Principal Investigator: Dr. Vo Duy Long

Institution: University Medical Center, University of Medicine and Pharmacy at Ho Chi Minh City

---

STUDY INFORMATION

Purpose of the Study

Gastric cancer is a major public health issue worldwide, with high morbidity and mortality rates. Surgical resection with D2 lymphadenectomy remains the standard treatment. In recent years, laparoscopic surgery has gained popularity due to its minimally invasive nature, leading to reduced blood loss, faster recovery, and less postoperative pain. However, the role of laparoscopic gastrectomy in T4a gastric cancer is still debated due to technical challenges and oncological concerns.

This study is conducted to compare the safety and efficacy of laparoscopic distal gastrectomy (LDG) versus open distal gastrectomy (ODG) in patients with T4a gastric cancer.

Study Objectives

- Compare the oncological outcomes between LDG and ODG, focusing on 3-year disease-free survival (DFS).
- Evaluate intraoperative and postoperative complications.

- Assess postoperative recovery and quality of life.

Study Design

- This is a randomized controlled trial (RCT).
- Total participants: 240 patients (120 in the LDG group, 120 in the ODG group).
- Patients will be recruited from the University Medical Center, Ho Chi Minh City from June 2020 to June 2025.
- Patients will be followed up for a minimum of 36 months.

ELIGIBILITY CRITERIA

Inclusion Criteria:

- Male or female, aged 18 to 80 years.
- Histologically confirmed T4a gastric cancer via endoscopy and CT scan.
- Good general health (ECOG performance status 0-1, ASA I-III).
- No evidence of distant metastases or bulky lymph nodes.
- Willingness to participate and signed informed consent.

Exclusion Criteria:

- Prior gastric surgery.
- Severe tumor-related complications (e.g., bleeding, perforation).
- Previous chemotherapy or radiotherapy.
- History of other malignancies in the past 5 years.
- Severe comorbidities that contraindicate surgery (e.g., heart failure, kidney failure).

STUDY PROCEDURE

Step 1: Screening

- Patients diagnosed with T4a gastric cancer will be assessed via endoscopy and CT scan.
- Eligible patients will be evaluated by a tumor board for confirmation.

Step 2: Informed Consent

- Patients will receive detailed information about the study, including procedures, risks, and benefits.
- Patients will sign the informed consent form before randomization.

Step 3: Randomization

- Patients will be randomly assigned to one of two groups:
    - Group 1: Laparoscopic distal gastrectomy (LDG) + D2 lymphadenectomy.
    - Group 2: Open distal gastrectomy (ODG) + D2 lymphadenectomy.
- Randomization will be conducted via computer-generated allocation.

Step 4: Surgery

- Surgery will be performed within 30 days of randomization.
- Both procedures follow Japanese Gastric Cancer Treatment Guidelines.

Step 5: Postoperative Care

- Standardized postoperative care for both groups, including:

   - Nutritional monitoring and pain management.
   - Wound and infection assessment.
   - Hospital discharge after 5–10 days, depending on individual recovery.

Step 6: Adjuvant Chemotherapy

- Patients with pathological Stage II or III disease will undergo adjuvant chemotherapy within 4–6 weeks post-surgery.

Step 7: Follow-up

- Regular follow-up visits at 3-month intervals for the first 2 years.
- Follow-up every 6 months for the next 3 years.
- Follow-up includes clinical assessment, CT scan, tumor markers (CEA), and additional imaging (PET-CT, MRI) if necessary.

BENEFITS & RISKS

Potential Benefits

- Patients receive standardized treatment and high-quality surgical care.
- The study may improve future treatment strategies for gastric cancer.

Potential Risks

- Surgical risks: Bleeding, infection, anastomotic leakage, pulmonary complications.
- Long-term complications: Bowel obstruction, hernia, chronic pain.
- No additional risks compared to standard treatment.

COST & COMPENSATION

- Patients will pay for treatment as per hospital policy.
- No additional costs will be charged for participating in the study.
- No financial compensation for participation.

CONFIDENTIALITY

- All personal and medical data will be kept confidential.
- Data will be used only for research purposes.

VOLUNTARY PARTICIPATION

- Participation is voluntary, and patients may withdraw at any time without affecting their medical care.

CONTACT INFORMATION

For further inquiries, please contact:

- Dr. Vo Duy Long – Phone: (+84) 918 133 915
- Dr. Tran Quang Dat – Phone: (+84) 905 621 107

CONSENT TO PARTICIPATE

I have read and understood the information above. I have had the opportunity to ask questions, and I have received satisfactory answers. I voluntarily agree to participate in this study.

Participant's Name: ___________________________
Signature: _________________________________
Date: _________________

WITNESS OR LEGAL REPRESENTATIVE (If applicable)

Witness/Legal Representative's Name: ___________________________
Signature: _________________________________
Date: _________________

INVESTIGATOR'S DECLARATION

I confirm that the participant has been fully informed about the study. The participant has signed the informed consent voluntarily.

Investigator's Name: ___________________________
Signature: _________________________________
Date: _________________

Appendix 3: Ethical approval by Institutional Review Board, University Medical Center Ho Chi Minh city

BỆNH VIỆN ĐẠI HỌC Y DƯỢC TPHCM
HỘI ĐỒNG ĐẠO ĐỨC TRONG NGHIÊN CỨU Y SINH HỌC

Số: 26 /GCN-HĐĐĐ

CỘNG HÒA XÃ HỘI CHỦ NGHĨA VIỆT NAM
Độc lập - Tự do - Hạnh phúc

Thành phố Hồ Chí Minh, ngày 11 tháng 6 năm 2020

GIẤY CHỨNG NHẬN
Chấp thuận của Hội đồng Đạo đức trong nghiên cứu y sinh học cơ sở

Căn cứ quyết định số 676/QĐ-BVĐHYD, ngày 26/3/2020 của Giám đốc Bệnh viện Đại học Y Dược TPHCM về việc thành lập Hội đồng Đạo đức trong nghiên cứu y sinh học cấp cơ sở;

Căn cứ biên bản họp ngày 06/5/2020 của Hội đồng Đạo đức trong nghiên cứu y sinh học Bệnh viện Đại học Y Dược TPHCM;

Trên cơ sở xem xét của thường trực Hội đồng Đạo đức trong nghiên cứu y sinh học Bệnh viện Đại học Y Dược TPHCM;

Hội đồng Đạo đức trong nghiên cứu y sinh học Bệnh viện Đại học Y Dược TPHCM chấp thuận về các khía cạnh đạo đức trong nghiên cứu đối với đề cương nghiên cứu:

1. Tên nghiên cứu: *Nghiên cứu so sánh ngẫu nhiên phẫu thuật nội soi với mổ mở cắt phần xa dạ dày và nạo hạch triệt để điều trị ung thư dạ dày giai đoạn T4a.*
2. Chủ nhiệm đề tài: TS BS. Võ Duy Long.
3. Địa điểm triển khai: Khoa Ngoại Tiêu hóa, Bệnh viện Đại học Y Dược TPHCM.
4. Thời gian thực hiện: 6/2020 – 6/2023.

Ngày chấp thuận: Ngày 11 tháng 6 năm 2020.

Những thay đổi trong triển khai nghiên cứu sẽ phải được Hội đồng Đạo đức trong nghiên cứu y sinh học Bệnh viện Đại học Y Dược TPHCM xem xét và chấp thuận, trừ trường hợp rõ ràng cần thiết thay đổi để loại trừ nguy cơ trực tiếp cho đối tượng nghiên cứu.

Nghiên cứu viên chính phải báo cáo các trường hợp biến cố bất lợi, biến cố bất lợi nghiêm trọng cho Hội đồng Đạo đức trong nghiên cứu y sinh học Bệnh viện Đại học Y Dược TPHCM theo đúng các quy định hiện hành.

Nơi nhận:
- Nghiên cứu viên chính;
- Đơn vị chủ trì;
- Lưu: HĐĐĐ.

CHỦ TỊCH HỘI ĐỒNG

Đặng Vạn Phước
