# Supplementary material for: Laparoscopic vs Open Distal Gastrectomy With D2 Lymphadenectomy for Clinical T4a Gastric Cancer: The UMC-UPPERGI-01 Randomized Clinical Trial
Source: JAMA Surg. 2025 Nov 12;161(1):9–18. doi: 10.1001/jamasurg.2025.4929 (PMC12613089; doi:10.1001/jamasurg.2025.4929)
Supplement: Supplement 2. — eTable 1. Inclusion and Exclusion Criteria eTable 2. Univariate and Multivariate Analysis for Predicting Postoperative Morbidity [file jamasurg-e254929-s002.pdf]

Supplemental Online Content

Dat TQ, Thong DQ, Nguyen DT, et al. Laparoscopic vs open distal gastrectomy with D2 lymphadenectomy for clinical T4a gastric cancer: the UMC-UPPERGI-01 randomized clinical trial. *JAMA Surg*. Published online November 12, 2025. doi:10.1001/jamasurg.2025.4929

eTable 1. Inclusion and Exclusion Criteria
eTable 2. Univariate and Multivariate Analysis for Predicting Postoperative Morbidity

This supplemental material has been provided by the authors to give readers additional information about their work.

© 2025 Dat TQ et al. *JAMA Surgery*.

eTable 1. Inclusion and Exclusion Criteria

**Inclusion criteria:**
(1) aged 18 to 80 years
(2) with histologically confirmed gastric adenocarcinoma located in the lower or middle third of the stomach
(3) staged as clinical T4aN0–3M0 based on preoperative imaging
(4) Eastern Cooperative Oncology Group (ECOG) performance status of 0 – 1
(5) American Society Anesthesiology physical status (ASA-PS) score of I – III
**Exclusion Criteria**
(1) pregnancy or breastfeeding women
(2) bulky lymph nodes
(3) tumor invaded to the duodenum
(4) previous gastric surgery
(5) severe tumor-related complications such as bleeding or perforation required to emergency surgery
(6) prior chemotherapy or radiotherapy
(7) receiving neoadjuvant chemotherapy
(8) other malignancies within the past 5 years
(9) severe conditions contraindicating laparoscopy.

© 2025 Dat TQ et al. *JAMA Surgery*.

eTable 2. Univariate and Multivariate Analysis for Predicting Postoperative Morbidity

		Univariate		Multivariate	
**Variables**	**Morbidity**	**OR (95% CI)**	**p-value**	**OR (95% CI)**	**p-value**
Age					
< 60	13/88 (14.8)	-			
≥ 60	32/120 (27.7)	2.28 (1.12, 4.64)	**0.040**	1.70 (0.79–3.65)	0.173
Sex					
Male	34/154 (22.1)	-			
Female	11/54 (20.4)	0.97 (0.46, 2.04)	0.939		
Aproach					
LDG	23/104 (22.1)	-			
ODG	22/104 (21.2)	0.85 (0.44, 1.63)	0.619		
BMI (kg/m2)					
< 25	35/145 (24.1)	-			
≥ 25	10/63 (15.9)	0.64 (0.30, 1.36)	0.183		
Comorbidity					
No	12/95 (12.6)	-			
Yes	33/113 (29.2)	3.10 (1.50, 6.41)	**0.004**	2.42 (1.11–5.30)	**0.026**
ASA					
1	1/9 (11.1)	-			
2	17/86 (19.8)	1.97 (0.23, 16.8)	0.535		
3	27/113 (23.9)	2.76 (0.33, 23.03)	0.348		
Tumor size					
< 5 cm	16/96 (16.7)	-			
≥ 5 cm	29/112 (25.9)	1.75 (0.88, 3.46)	0.110	1.46 (0.71–2.98)	0.300
Albumin					
<35	6/25 (24.0)	-		-	
≥ 35	39/183 (21.3)	0.86 (0.32, 2.29)	0.760		
Anemia					
No	27/146 (18.5)	-			
Yes	18/62 (29.0)	1.80 (0.90, 3.56)	0.094	-	
GOO				-	
No	35/148 (23.7)	-		-	
Yes	10/60 (16.7)	0.65 (0.30, 1.41)	0.270	0.69 (0.31–1.54)	0.363
pT					
pT1-pT3	12/64 (18.8)	-			
pT4a	33/144 (22.9)	1.29 (0.62, 2.70)	0.501		
pN					
N0	10/51 (19.6)	-		-	
N1-2	16/83 (19.3)	0.98 (0.41, 2.36)	0.963		
N3	29/74 (25.7)	0.431 (0.60, 3.37)	0.431		
Type of anastomosis					
Billroth II	33/163 (20.2)	-			
Rouxx-En-Y	12/45 (26.7)	1.43 (0.67, 3.07)	0.356		

Statistic is n (%)
CI: confidence interval; OR: Odds ratio; LDG: laparoscopic distal gastrectomy
ODG: open distal gastrectomy; GOO: Gastric outlet obstruction

© 2025 Dat TQ et al. *JAMA Surgery*.
