# Supplementary material for: Laparoscopic vs Open Distal Gastrectomy With D2 Lymphadenectomy for Clinical T4a Gastric Cancer: The UMC-UPPERGI-01 Randomized Clinical Trial
Source: JAMA Surg. 2025 Nov 12;161(1):9–18. doi: 10.1001/jamasurg.2025.4929 (PMC12613089; doi:10.1001/jamasurg.2025.4929)
Supplement: Supplement 3. — Data Sharing Statement [file jamasurg-e254929-s003.pdf]

Data Sharing Statement

Dat. Laparoscopic vs Open Distal Gastrectomy With D2 Lymphadenectomy for Clinical T4a Gastric Cancer. *JAMA Surg*. Published November 12, 2025. doi:10.1001/jamasurg.2025.4929

Data
Additional Information: ClinicalTrials.gov Identifier: NCT04384757
Data available: Yes
Data types: Deidentified participant data
How to access data: Contact to corresponding author: long.vd@umc.edu.vn
When available: With publication

Supporting Documents
Document types: Statistical/analytic code, Informed consent form
How to access documents: Contact to corresponding author: long.vd@umc.edu.vn
When available: With publication

Additional Information
Who can access the data: researchers whose proposed use of the data has been approved
Types of analyses: researchers whose proposed use of the data has been approved
Mechanisms of data availability: with investigator support
Any additional restrictions: For commercial reason
